# Trade-offs between Sustainable Development Goals in carbon capture and utilisation†

**DOI:** 10.1039/d2ee01153k

**Published:** 2022-08-31

**Authors:** Iasonas Ioannou, Ángel Galán-Martín, Javier Pérez-Ramírez, Gonzalo Guillén-Gosálbez

**Affiliations:** a Institute for Chemical and Bioengineering, Department of Chemistry and Applied Biosciences, ETH Zürich Vladimir-Prelog-Weg 1 8093 Zürich Switzerland jpr@chem.ethz.ch gonzalo.guillen.gosalbez@chem.ethz.ch; b Department of Chemical, Environmental and Materials Engineering, Universidad de Jaén Campus Las Lagunillas s/n 23071 Jaén Spain; c Center for Advanced Studies in Earth Sciences, Energy and Environment. Universidad de Jaén Campus Las Lagunillas s/n 23071 Jaén Spain

## Abstract

Carbon capture and utilisation (CCU) provides an appealing framework to turn carbon emissions into valuable fuels and chemicals. However, given the vast energy required to activate the CO_2_ molecule, CCU may have implications on sustainable development that are still poorly understood due to the narrow scope of current carbon footprint-oriented assessments lacking absolute sustainability thresholds. To bridge this gap, we developed a power-chemicals nexus model to look into the future and understand how we could produce 22 net-zero bulk chemicals of crucial importance in a sustainable manner by integrating fossil, CCU routes and power technologies, often assessed separately. We evaluated the environmental performance of these technologies in terms of their contribution to 5 Sustainable Development Goals (SDGs), using 16 life cycle assessment metrics and 9 planetary boundaries (PB) to quantify and interpret the impact values. We found that fossil chemicals could hamper the attainment of SDG 3 on good health and well-being and SDG 13 on climate change. CCU could help meet SDG 13 but would damage other SDGs due to burden-shifting to human health, water scarcity, and minerals and metals depletion impacts. The collateral damage could be mitigated by judiciously combining fossil and CCU routes with carbon-negative power sources guided by optimisation models incorporating SDGs-based performance criteria explicitly. Our work highlights the importance of embracing the SDGs in technology development to sensibly support the low-carbon energy and chemicals transition.

## Abstract 2

We analyse the future transition towards a carbon-neutral chemical sector. Our study unfolds new avenues to include SDG-based metrics in science and engineering while quantifying the potential collateral damage of CCU on sustainable development.

## Graphical abstract

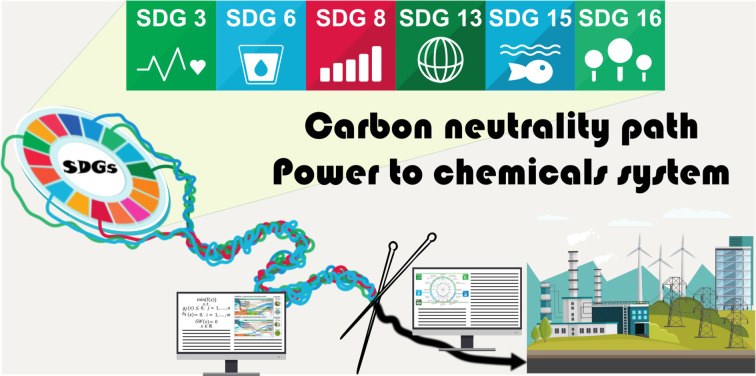

**Broader context**

Combatting climate change is currently driving sustainable technology development. However, emerging technologies, like carbon capture and utilisation (CCU), may have substantial repercussions on our socio-economic systems and the environment beyond global warming. In 2015, the United Nations put forward 17 Sustainable Development Goals (SDGs) to measure the progress made towards sustainable development. These goals are often evaluated at the national level, while studies applying them to the assessment of emerging low-carbon technologies are very scarce and often qualitative. Here we apply for the first time the SDGs framework to quantitatively evaluate the broad implications of transforming CO_2_ into key chemicals on sustainable development, elucidating whether efforts to meet the climate action SDG 13 could hamper other SDGs due to burden-shifting. Our large-scale analysis of CCU, which explicitly models the power-chemicals nexus and uses 9 PBs to contextualise the SDGs performance, underscores the importance of evaluating impacts beyond SDG 13 on climate action to avoid myopic solutions eroding our ability to live sustainably. Overall, this study unfolds new avenues to include SDG-based metrics in quantitative assessments in science and engineering while quantifying the potential collateral damage of CCU on sustainable development.

## Introduction

The United Nations (UN) introduced in 2015 the 2030 Agenda for Sustainable Development, which reflects the collective views of a desirable future and addresses the main challenges humanity faces. These include ending poverty and other deprivations, improving health and education, reducing inequality, and spurring economic growth, all this while combatting climate change and preserving our ecosystems. The Agenda, supported by the 193 UN member states, covers 17 Sustainable Development Goals (SDGs), 169 internationally agreed targets and 232 indicators,^1^ among which climate change and biodiversity loss are attracting growing attention.^2^

Technology development for sustainable energy and chemicals provision currently focuses primarily on combatting climate change, which falls within the realm of SDG 13 (climate change action). Accordingly, emerging technologies are often only assessed in terms of their carbon footprint, quantified following life cycle assessment principles (LCA).^3–6^ These widespread LCA studies omit impacts beyond climate change or evaluate them *via* indicators that lack impact thresholds, making them hardly interpretable from a global sustainability viewpoint.^3,7,8^ Consequently, the extent to which future low-carbon technological roadmaps improving SDG 13 could contribute to other dimensions of sustainable development remains unclear because their performance in other SDGs, often evaluated at the national rather than at the technological level,^9,10^ is overlooked.

Here we argue that SDGs-based assessments of emerging technologies are critical to avoid low-carbon pathways shifting burdens across sustainability dimensions. With this spirit, we focus on studying the broad implications of carbon capture and utilisation (CCU) – a growing trend in low-carbon fuels and chemicals production – on the SDGs attainment. CCU could help curb emissions while creating economic value,^11^ yet its vast energy requirements are today a major obstacle.^12^ Notably, the increase in energy demand will require skilled labour for the installation and operation of the power system,^13,14^ which will impact SDG 4 (quality education) and 8 (decent work and economic growth), respectively. Substantial ongoing research focuses on electro- and thermocatalytic routes converting CO_2_ into a range of chemicals and fuels, including methanol, formic acid, and ethylene, among others.^15^ Extensive literature evaluated CCU routes focusing on single technologies or a small subset of them,^15–18^ omitting their links with the power mix and applying conventional LCA metrics limited in scope, *e.g.*, carbon footprint and fossil resources depletion.^7,19,20^ However, recent qualitative studies highlighting trade-offs between SDGs in CCU,^21^ also found in food^22^ and energy^23^ systems, and micro-plastics,^24^ stress the importance of measuring the potential undesired adverse effects of CCU on the SDGs attainment, as we do here.

Until recently, the lack of suitable metrics prevented the use of SDGs in technology-oriented assessments. However, the recent concept of absolute sustainability,^25^ which allows incorporating the planet's carrying capacity into LCA-based PBs indicators, has unfolded new avenues to perform PBs-SDGs studies^26^ of emerging technologies. Notably, LCA approaches based on absolute sustainability have started to emerge in past years,^27,28^ most of them building on the works by Rockstrom *et al.* (2009)^29^ and Steffen *et al.* (2015),^30^ who defined 9 planetary boundaries (PBs) for humanity to operate the planet safely. These LCA-PB-based assessments provide a reference to interpret the system's performance considering the Earth's carrying capacity,^31^ which allows contextualising the LCA results from an absolute sustainability and SDGs perspective. Along these lines, Sala *et al.*^25^ introduced an LCA-PB-based assessment that combines the Environmental Footprint (EF) LCA method adopted at the EU level^32^ and the updated LANCA model^33,34^ with the SDGs framework. This approach essentially mapped 16 LCA impact categories to 5 SDGs and 9 PBs, incorporating absolute sustainability concepts in decision-making. Despite these advances, to our best knowledge, SDG-based methods were never applied to assess low-carbon technologies, including CCU.

**Fig. 1:**
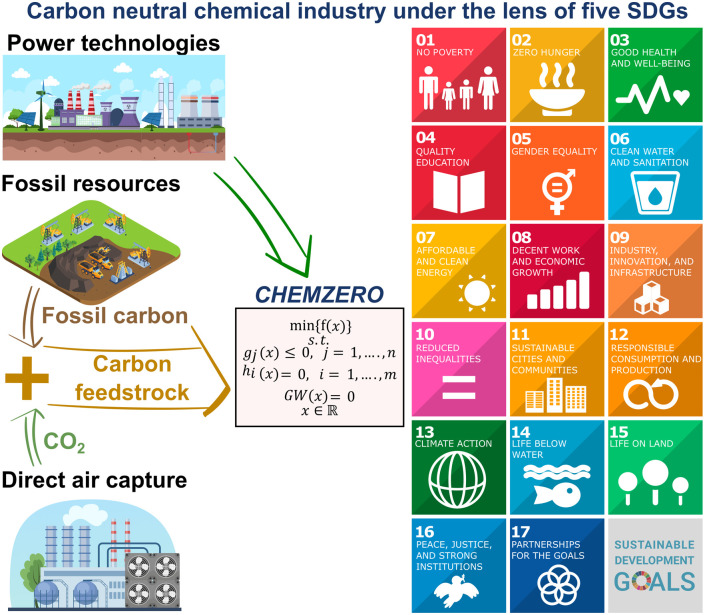
*CHEMZERO* identifies optimal low-carbon roadmaps for the chemical industry by integrating power and chemical production technologies. The model includes the LCA impact categories of the Environmental Footprint (EF) method, which are connected to 5 SDGs and 9 PBs *via* an LCA-based PBs-SDGs approach.^25^ The LCA-based PBs-SDGs method considers 5 SDGs, SDG 3 (good health and well-being), 6 (clean water and sanitation), and 13–15 (climate action, life below water, and life on land, respectively). These SDGs are closely linked to standard LCA indicators, as opposed to others with weaker links to engineering decisions and inherently more qualitative.

Here we capitalise on absolute sustainability methods to expand our limited knowledge of how deploying CCU on a large scale could affect the attainment of SDG 8 and a set of 5 SDGs that can be directly linked to the system's economic and environmental performance, respectively. For this purpose, we built a model of the future chemical industry integrating fossil and CCU routes (Fig. 1) with a tailored power mix to identify carbon-neutral roadmaps according to different criteria and constraints (related to several SDGs) to minimise the system's (i) total cost or (ii) overall transgression level. We found that cost-effective CCU solutions to attain carbon neutrality in 2050 could hamper our ability to meet several SDGs. However, this collateral damage could be mitigated by judiciously combining fossil and renewable-carbon technologies to maximise the SDGs performance. Overall, our analysis highlights the need to incorporate the SDGs into decision-making for technology development and meet SDG 13 on climate change in a more sustainable way.

## Materials and methods

To carry out our analysis, we developed a network model capturing the interplay between the chemical and power sectors, named *CHEMZERO*, that identifies carbon-neutral (on a cradle-to-gate basis, that is, from raw materials acquisition to chemicals production, omitting the end-use phase) pathways for chemicals production optimising either the cost or a transgression performance metric connected to the SDGs, *CHEMZERO*_*cost*_ and *CHEMZERO*_*sust*_, respectively. Notably, unlike other models that omit the power-chemicals nexus,^7^*CHEMZERO* jointly optimises power and chemical production technologies to satisfy the future demand of 22 major chemicals in 2050, representing most of the chemical industry's energy demand and GHG emissions.^35^ Hence, the model automatically identifies the main conversion pathways from a set of available technologies in order to optimise given criteria while not violating a set of technical and market-related constraints. The model and LCA calculations are outlined next, while further details are available in the ESI.†

### Network modelling and mathematical optimisation

**Fig. 2:**
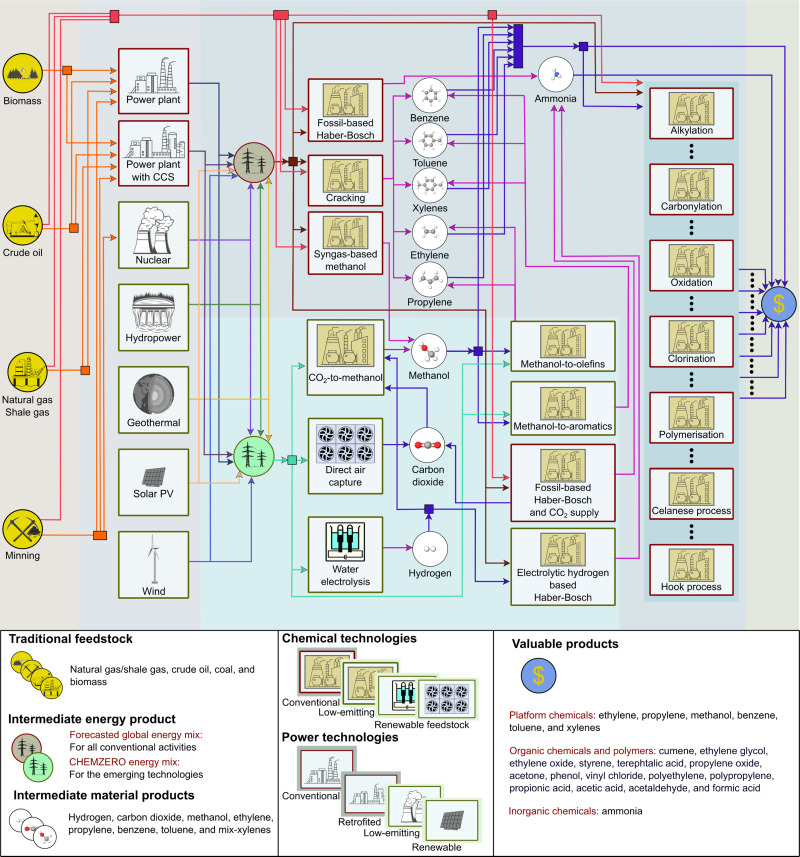
Superstructure of the *CHEMZERO* network considered in this work. Several energy technologies can be judiciously integrated to design a power system for *CHEMZERO* (left). The bespoke mix covers the energy needs of a subset of processes and provides co-generated heat from CHP power plants. This mix powers electrolytic H_2_, CO_2_ capture (*via* direct air capture), and the CO_2_-to-methanol (MeOH), methanol-to-olefins (MTO), and methanol-to-aromatics (MTA) processes. The chemicals’ final demand is met by integrating conventional fossil-based and CCU processes (right) to attain the minimum (i) total cost or (ii) overall transgression level of the system.

*CHEMZERO* satisfies the chemicals demand by selecting (i) conventional fossil pathways, (ii) CCU technologies, or (iii) a combination of both (simplified superstructure in Fig. 2), all of them regarded as mature technologies with high technology readiness levels (TRL ≥ 7). CCU technologies convert CO_2_ and electrolytic hydrogen (eH_2_) into green methanol (MeOH), which is subsequently processed into other chemicals through the methanol-to-olefins and -aromatics routes (MTO and MTA, respectively). We also consider three Haber–Bosch (HB) configurations for producing ammonia, *e.g.*, (i) the conventional HB using natural gas (NG) steam reforming (SR-HB); (ii) SR-HB coupled with CCU to capture and utilise the CO_2_ emitted by SMR H_2_ (SR-HB-CCU); and (iii) eH_2_ based HB (known as green HB), which replaces the SMR H_2_ by electrolytic H_2_ (eHB). The mass and energy balances defined for the above technologies, among which the model will identify the optimal ones according to some criteria, are based on information from process simulations, databases,^36^ and other literature sources (see Section S1.2 of the ESI† for more details).

The CO_2_ raw material for CCU can be sourced from SR-HB^37,38^ (at 25.0 $ t^−1^) using a CO_2_ stream often consumed in urea production,^39^ or from direct air capture (DAC) by implementing Climeworks’ technology projected to 2050.^40^ Furthermore, *CHEMZERO* concurrently optimises the power mix linked to the chemical system following previous work by some of us.^41^ The bespoke energy system, which powers the eH_2_, DAC, MeOH, MTO, and MTA, includes both (i) state-of-the-art power technologies and (ii) combined heat and power (CHP) facilities. The system's energy availability is ensured by balancing both intermittent and firm technologies.^42^

Producing eH_2_ requires substantial resources, *e.g.*, minerals and metals, which might become scarce,^43^ constraining the technologies’ potential and deployment. Hence, our model constrains the gradual deployment of power technologies using diffusion rates to capture exogenous factors limiting the speed of deployment (*e.g.*, market forces or social acceptance barriers) and considers resource availability constraints (related to SDG 7). The power technologies considered include oil, coal, natural gas, and bioenergy with and without carbon capture and storage (BECCS), wind (onshore and offshore), solar (photovoltaic, PV), geothermal, nuclear, and hydropower reservoirs. We provide in Section S1.2 of the ESI† information further details on the latter generation technologies. BECCS, alone or integrated with CHP,^44^ can deliver carbon-negative electricity but requires geological storage capacity to store the CO_2_ permanently. Furthermore, we assume that CHP plants could satisfy the heat demand (*i.e.*, steam) of the industrial processes and DAC, while purge streams from MeOH production could provide extra heat,^17,45^ diminishing the natural gas requirements (aligned with SDG 12).

*CHEMZERO* is formulated as a linear programming (LP) model that seeks pathways towards carbon neutrality subject to various technical, costs and impact-related constraints (eqn (1)). Hence, the model reflects the ambition of making the chemical industry carbon-neutral by the first half of the 21st century, as supported by the European Chemical Industry Council.^46^ This overall goal could be met at minimum cost by solving the model below.1
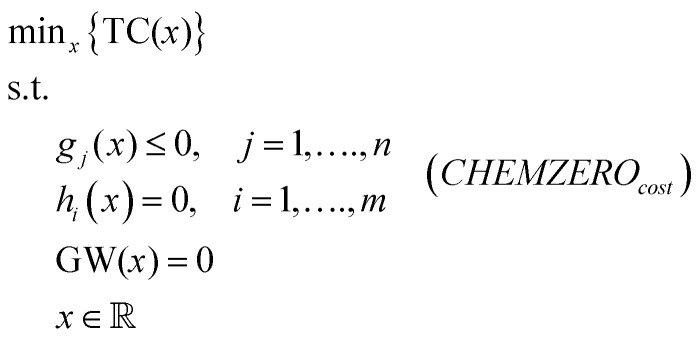
where *x* denotes continuous variables representing mass and energy flows, TC is the total cost, *g*_*j*_ and *h*_*i*_ are inequality and equality constraints, respectively, and GW is the life cycle global warming potential. *CHEMZERO*_*cost*_ overlooks environmental impacts beyond climate change. Alternatively, the model can attempt to reach carbon neutrality while minimising a sustainability metric linked to the SDGs (instead of the cost), which is quantified using an LCA-based PBs-SDGs framework.^25^ Accordingly, we define an alternative model, termed *CHEMZERO*_*sust*_, that minimises a transgression-based sustainability metric quantified following LCA principles, subject to the same constraints as before:2
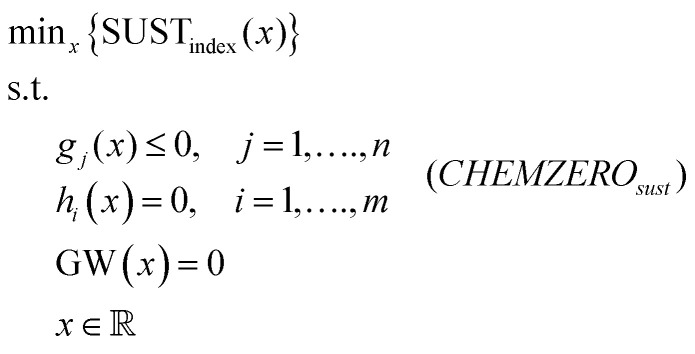
The ensuing sections describe how the LCA metrics are quantified and connected to the SDGs to evaluate the sustainability performance of the chemical system.

### Life cycle assessment

We apply the LCA methodology following the ISO 14040 and 14044 standards^47,48^ to ultimately measure the performance of the chemical technologies in 5 SDGs, as described next.

### Goal and scope definition

We quantify the impact of the chemical system relative to 9 PBs connected to 5 SDGs, considering as the functional unit the global demand for chemicals in 2050 (see ESI†). We adopt a cradle-to-gate scope where the system boundaries encompass the water splitting, MeOH, MTO, MTA, and DAC processes, the facilities further transforming the platform chemicals, and the surrounding activities supplying inputs to the foreground system (*i.e.*, background system), including the power system covering its energy needs.

### Life-cycle inventory

The life cycle inventories are obtained by combining data of the (i) foreground system (*i.e.*, energy mix, water splitting, DAC, and chemical technologies) and (ii) background system. Type (i) data are modelled using ecoinvent v3.5^36^ complemented with literature data, *i.e.*, water-splitting membrane,^49^ DAC,^40^ HB processes,^49^ and MeOH,^17^ MTO,^50^ and MTA^51^ facilities. In contrast, all flows of type (ii) are retrieved from ecoinvent v3.5^36^ accessed *via* SimaPro version 9.1.0.11.

### Life cycle impact assessment

We quantify 16 LCA indicators *via* the LCA method EF 2.0, recommended by the European Commission (2013/179/EU),^32^ while evaluating the land use indicator using the LANCA method.^33,34^ Following Sala *et al.*,^25^ the 16 LCA indicators were mapped to 5 SDGs (*i.e.*, 3, 6, 13–15) using 9 PBs to evaluate the severity of the impact. As discussed next, this severity is given by the ratio between the impact value and the maximum allowable impact dictated by the PBs.

### SDGs performance: the LCA-PBs-based objective function

We define an aggregated sustainability index based on the transgression level in each LCA indicator, where all indicators are given equal weights, as follows:3
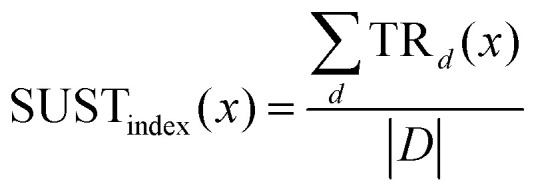
Here, TR_*d*_ is the transgression level of LCA indicator *d*, as defined next, and |*D*| denotes the total number of metrics (*i.e.*, the cardinality of set *D*). A system is sustainable if it lies within the maximum allowable impact in all the indicators, *i.e.*, TR_*d*_ is less than one in all the metrics. Otherwise, it should be deemed unsustainable. TR_*d*_ is calculated based on the system's total impact, TIMP_*d*_, and maximum carrying capacity, SOS_*d*_ (*i.e.*, safe operating space, SOS, given by the PB, denoting the ecological budget that should not be exceeded by all anthropogenic activities jointly^28^), as given in eqn (4):4
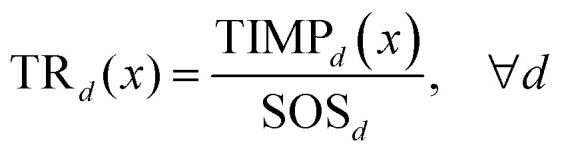
Moreover, Sala and co-workers defined a fixed zone of uncertainty for the carrying capacities used in their method.^25^ Accordingly, the SOS is defined as the area within the carrying capacity, the zone of uncertainty lies between one time the carrying capacity and twice its value, while the high-risk zone corresponds to impacts beyond twice the carrying capacity. We follow a precautionary approach based on the SOS limit, although a less conservative approach could be adopted by considering twice the values of the carrying capacities when estimating the transgression levels.

To draw meaningful conclusions, we study both the aggregated indicator SUST_index_ and the transgression level in each individual metric, taking the Earth's carrying capacity as a reference to interpret the results from an absolute sustainability viewpoint. As discussed later, these transgression levels are employed as a proxy for SDGs performance.

### Avoidance cost

CCU routes are often economically unappealing compared to their fossil-based counterparts. However, taxation on greenhouse gas (GHG) emissions could make them competitive. To explore the economic feasibility of mitigating climate change *via* CCU, we compute the avoidance cost (AC) of each solution as follows:5
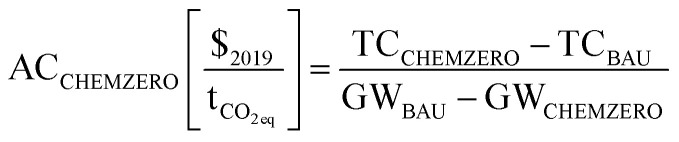
where TC_BAU/CHEMZERO_ and GW_BAU/CHEMZERO_ represent the total cost and global warming potential of the chemical system based on the *BAU* (business as usual) and *CHEMZERO* (optimal solution) configurations, respectively. Similarly, an avoidance cost can be computed for each chemical *i* (AC_*i*_) as follows:6
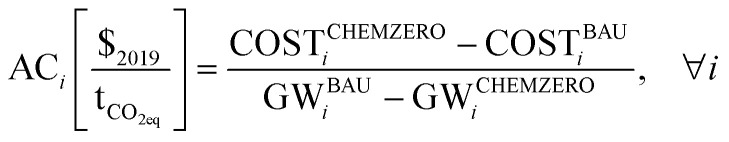
where COST^BAU/CHEMZERO^_*i*_ and GW^BAU/CHEMZERO^_*i*_ denote the cost and global warming potential of chemical *i* based on the *BAU*/*CHEMZERO* configuration, respectively. Note that AC_*i*_ is linked to chemical *i*'s final demand, cost, and mitigation potential, where the latter two depend on the synthesis route.

## Results and discussion

**Fig. 3:**
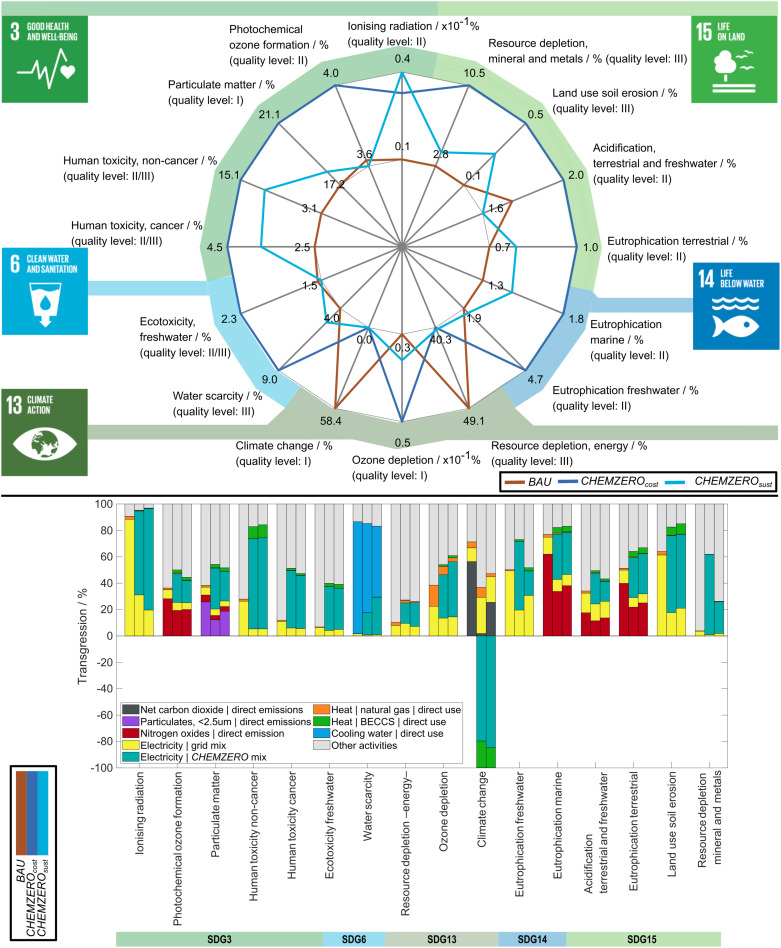
Transgression of the total safe operating space (SOS, *i.e.*, maximum limit defined on the LCA metrics) for the *BAU* (brown area), *CHEMZERO*_*cost*_ (blue area) and *CHEMZERO*_*sust*_ (cyan area) solutions (top), and breakdown of impacts for the latter systems (bottom). The mapping of the LCA indicators and the SDGs of human health (SDG 3), clean water and sanitation (SDG 6), climate change (SDG 13), life below water (SDG 14), and life on land (SDG 15) is based on the work by Sala *et al.*^25^ Furthermore, the environmental breakdown is presented based on 9 contributors, namely (1) net CO_2_ emissions, (2) particulate matter (<2.5 μm) emissions, (3) nitrogen oxides emissions – where (1), (2), and (3) are emitted directly in the chemical processes – (4) grid electricity – consumed directly in the conventional chemical processes – (5) electricity from the *CHEMZERO* mix, mainly to generate eH_2_, (6) heat from natural gas, (7) heat from BECCS – both (6) and (7) are consumed directly in the chemical processes and DAC – (8) cooling water – consumed directly in the chemical processes – and (9) other activities, which include inputs of fossil-based resources (or inputs of other nature), and direct emissions to air and water that are not covered in the previously mentioned categories. The environmental impacts are classified according to their quality level as recommended by the European Commission's Joint Research Centre.^53,54^

We first discuss the impact of the model solutions on the SDGs attainment, then describe their technological features, and finally study their financial implications. In evaluating the impact values on the SDGs, we consider the PBs as a reference. Hence, high transgression levels, *e.g.*, >10% (reference value discussed in the ESI†), would make it very challenging for the global economy as a whole to operate within the total ecological budget due to the high impact of the chemicals considered here. We note that some PB values can be controversial due to unclear ecosystem responses, global aggregate values, or knowledge gaps.^29,52^ Therefore, when interpreting the results, we pay special attention to identifying general patterns and trends and understanding the main differences across scenarios. At the same time, we consider the LCA indicators' quality level, as recommended by the European Commission's Joint Research Centre (see Fig. 3 labels and Section S1.4 of the ESI†).^53,54^

### Burden-shifting across SDGs

**Fig. 4:**
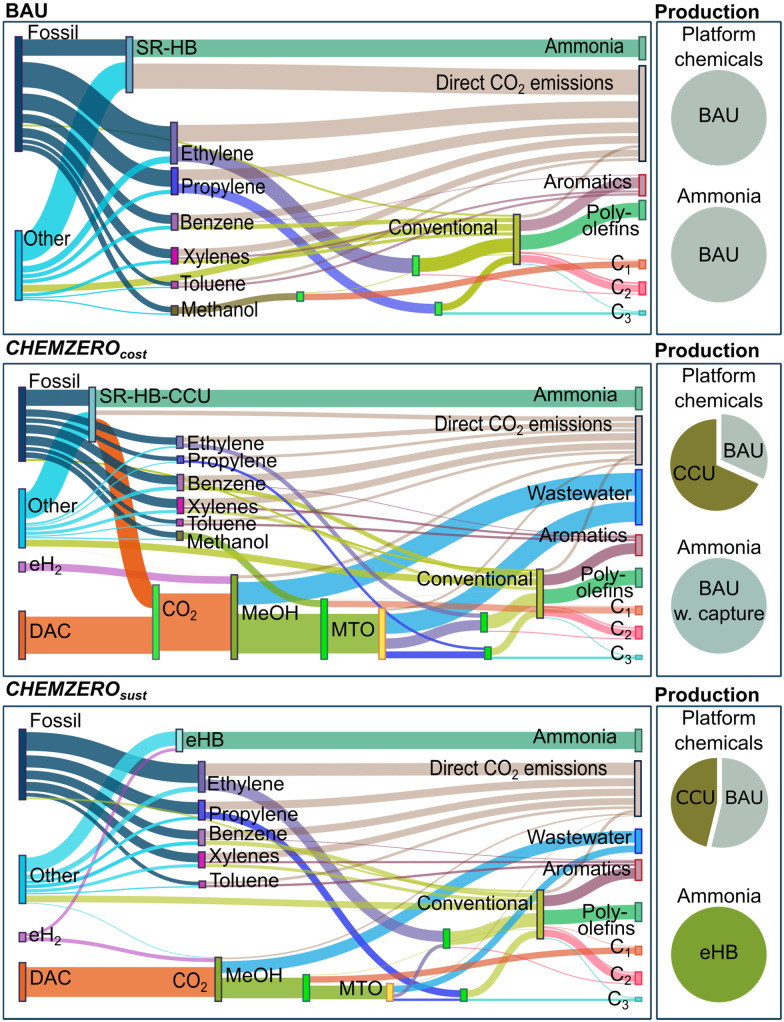
Total mass flows, gate-to-gate, within the chemical system of the *BAU* and of the two optimal solutions. The objective function influences the technologies’ selection drastically. Both solutions reduce the mass of fossil-based feedstock (*e.g.*, oil, coal, natural gas, and shale gas) and the direct CO_2_ emissions compared to the *BAU*. Furthermore, the optimal solutions offset the fossil-based emissions by utilising carbon-negative electricity (embodied in the eH_2_) and CO_2_ from air.

We start by studying the current fossil-based *BAU* chemical industry and whether CCU pathways would cause any collateral damage when attempting to curb carbon emissions. Focusing on the *BAU* (Fig. 3 – top – brown area), we find that using only fossil carbon as feedstock in chemicals production (Fig. 4) clearly hampers attaining SDGs 3 and 13. This is because fossil chemicals occupy a large percentage of the SOS in the climate change and resource depletion – energy – indicators, both linked to SDG 13. Here, we note that the quantification of the climate change metric has a low uncertainty level (quality level I), while the level of uncertainty in energy depletion is high (quality level III). Notably, the *BAU* solution consumes 58.4% of the maximum global allowable impact in climate change because it emits 4.0 Gt_CO_2eq__ year^−1^ on a life cycle basis while the PB equals 6.8 Gt_CO_2eq__,^25^ leaving little room for the other sectors to operate within this limit. This impact is followed by 49.1% in energy resource depletion (SDG 13) and 17.2% in particulate matter (quality level I), linked to SDG 3. The overall average transgression across all metrics, SUST_index_, is 9.2.

Moving to the CCU routes, we find that *CHEMZERO*_*cost*_ improves the climate change metric and the aggregated index greatly (SUST_index_ = 7.3), yet, it leads to significant burden-shifting. Notably, this solution worsens 14 out of the 16 LCA indicators (Fig. 3 – top – blue area), some of them quite substantially, which highlights the perils of myopic cost-effective climate change mitigation strategies. Notably, *CHEMZERO*_*cost*_ would make it easier to fulfil SDG 13, but still hamper attaining SDG 3 while also exerting significant pressure on SDGs 6 and 15, seemingly non-critical in the *BAU* solution (<4.0%). Here, the climate change impact would be zero as imposed in the model (GHG neutrality). However, because this solution would consume fossil resources and a high amount of energy, it would still show a significant transgression in resource depletion – energy – (40.3%). Arguably, this should not be of concern, as CCU and carbon-negative electricity would offset (on a cradle-to-gate basis) the carbon emissions linked to fossil resources usage, as discussed later, while the environmental damage is 17.9% lower than the *BAU*. This transgression level is followed by particulate matter (21.1%, quality level I), non-cancer human health effects (15.1%, quality level II/III – medium-to-high uncertainty), both linked to SDG 3, resource depletion – mineral and metals – (10.5%, quality level III), related to SDG 15, and water scarcity (9.0%, quality level III), connected to SDG 6.

The *CHEMZERO*_*sust*_ solution (Fig. 3 – top – cyan area) alleviates the collateral damage substantially, improving *CHEMZERO*_*cost*_ in 13 indicators simultaneously, attaining the best SUST_index_ (5.9 *vs.* 7.3 and 9.2); however, it outperforms the *BAU* in only 5 metrics. SDGs 6 and 15 would become much less critical in this solution due to the improvements in resource depletion – mineral and metals – (4.1% *vs.* 10.5%, quality level III), and water scarcity (5.1% *vs.* 9.0%, quality level III), but meeting SDGs 3 would still be challenging due to its high non-cancer human toxicity (quality level II/III) and particulate matter (quality level I) impact (11.5% and 18.1%, respectively).

**Fig. 5:**
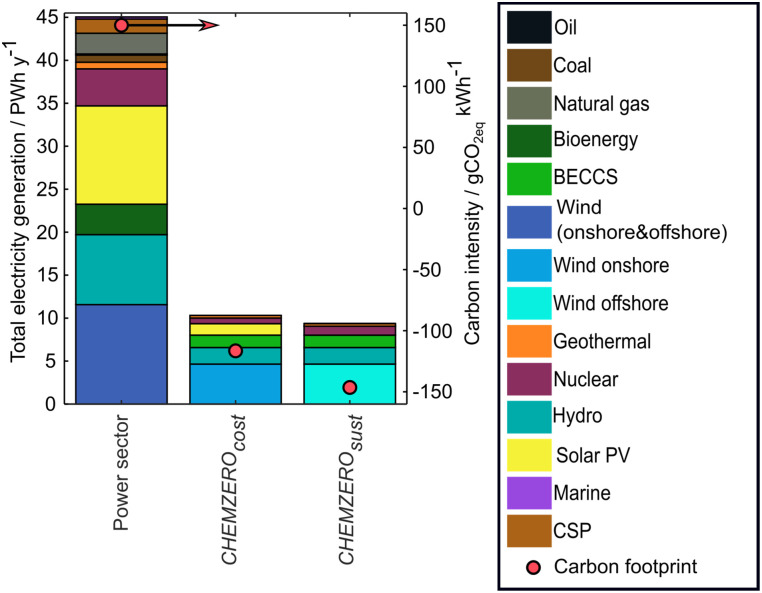
Annual power generation and breakdown for the global anthropogenic activities in 2050, as forecasted in the world energy outlook,^38^ and power generation from the bespoke mixes of the *CHEMZERO*_*cost*_ and *CHEMZERO*_*sust*_ (primary axis) their respective carbon footprint (secondary axis).

The impact breakdown (Fig. 3 – bottom) reveals that many damage categories worsen when deploying CCU because of its large energy requirements. While both CCU solutions consume vast amounts of power, *CHEMZERO*_*sust*_ often outperforms *CHEMZERO*_*cost*_ due its lower energy consumption, the shift from onshore to offshore wind turbines, and the avoidance of solar panels (Fig. 5, further details on the power mix impacts in Table S7 and Fig. S1 of the ESI†). Focusing on the individual SDGs, particulate matter (SDG 3), critical in all the solutions, is linked in the *BAU* to the lumped term other activities, followed by the direct release of particulates – below 2 μm – and NO_*x*_ from the chemical processes and power and heat consumption. In the alternative CCU solutions, the contribution of the power mix is also very significant. The latter causes most of the impact in the human toxicity non-cancer category, which is particularly severe in the *CHEMZERO*_*cost*_ solution. Water scarcity (SDG 6), mostly linked to the evaporation losses from the cooling towers in the *BAU*, worsens in the alternative solutions due to the additional MeOH plants requiring substantial cooling and the hydropower facilities in the power system (Fig. 3, 4 and Fig. S1, ESI†). Climate change (SDG 13) is mostly linked to direct fossil CO_2_ emissions and power and heat supply in the *BAU*, while these emissions are offset in the carbon-neutral solutions by consuming DAC CO_2_ and carbon-negative electricity and heat. Finally, resource depletion – mineral and metals – (SDG 15) worsens in the CCU solutions due, again, to the contribution of the bespoke mix (Table S7, ESI†).

### Hybridisation of fossil and CO_2_-based chemical routes

To attain carbon neutrality, the CCU roadmaps in *CHEMZERO*_*cost*_ and *CHEMZERO*_*sust*_ integrate fossil and renewable-carbon-based technologies, exploiting synergies between them. Both solutions differ substantially in the shares of fossil and CCU chemicals, the ammonia process configuration, and the CO_2_ provenance (Fig. 4).

The BAU solution produces ammonia *via* SR-HB, and the platform chemicals (ethylene, propylene, benzene, toluene, and xylenes) through cracking, subsequently transforming them into 15 chemicals *via* conventional routes. In contrast, *CHEMZERO*_*cost*_ uses CO_2_ captured in the Haber–Bosch plants and from the air (0.6 and 1.2 Gt_CO_2__, respectively) to produce green MeOH and convert it into olefins *via* MTO. This solution increases the annual production of methanol by 6.7-fold relative to the *BAU*, where the majority is produced from the green MeOH process. MTO plants convert most of this methanol into ethylene and propylene, whose demand is mainly covered by the methanolic route (∼70%). Conversely, aromatics are entirely produced *via* fossil-based cracking since the MTA process is not selected. 231.7 Mt of eH_2_ are consumed exclusively for green methanol synthesis, and a significant amount of it is lost in the MeOH and MTO processes due to the wastewater by-product.

*CHEMZERO*_*sust*_ makes less use of CCU and deploys the green HB process. This solution substantially reduces the amount of methanol produced (from 1433.0 to 636.9 Mt) and the capacity of the MTO process, fully operated with green MeOH. It generates most of the ethylene and propylene from fossil carbon (>75%) and produces all the ammonia *via* the green HB process, avoiding the CO_2_ by-product and consuming 43.7% of the 215.0 Mt of eH_2_ (the rest used for green MeOH synthesis). The carbon feedstock is entirely sourced from DAC plants (0.9 Gt), while the MTA process is again omitted. Hence, using carbon-negative electricity for green ammonia synthesis allows attaining carbon neutrality despite increasing the amount of fossil olefins and reducing the DAC capacity. Finally, the quantity of the co-produced wastewater is reduced significantly.

### Carbon-negative power to produce carbon-neutral chemicals

Both CCU roadmaps rely on carbon-negative power (Fig. 5) to generate carbon-negative eH_2_ and offset other carbon emissions, ultimately attaining carbon-neutrality (−1.2 × 10^−1^ to −1.5 × 10^−1^ t_CO_2eq__ MW h^−1^, resulting in eH_2_ and CO_2_ feedstock with a carbon intensity, respectively, of −4.8 to −6.1 t_CO_2eq__ t_H_2__^−1^ and −0.8 to −1.3 t_CO_2eq__ t_CO_2__^−1^, respectively). Moreover, the minimum cost and transgression level power systems mainly differ in the amount of electricity produced, a shift from onshore to offshore turbines, and the solar and nuclear power capacities (Fig. 5). CCU would require 10.3 and 9.4 PW h of power, respectively, primarily for eH_2_ production (93.8–95.9%, details in the Fig. S1, ESI†), where BECCS, hydropower – reservoir – nuclear, and geothermal facilities would provide the firm power required to support the significant penetration of intermittent wind and solar. This high electricity consumption represents an additional 22.9–20.8% power relative to the forecasted global generation in 2050.^38^ In *CHEMZERO*_*cost*_, wind power would show the highest share, followed by hydropower, BECCS, solar PV, nuclear, and geothermal. In contrast, *CHEMZERO*_*sust*_ would not rely on solar PV and would almost double nuclear power generation. Moreover, in both systems, BECCS would generate both power (1.4 PWh) and heat (5.0–4.1 EJ), making the energy inputs carbon negative due to the storage of biogenic carbon (Fig. S1, ESI†). In both solutions, DAC would become the primary steam consumer from the BECCS–CHP plants, using 90.6–88.8% of the total carbon-negative steam.

### CCU mitigation *vs.* NETs removal for curbing carbon emissions

**Fig. 6:**
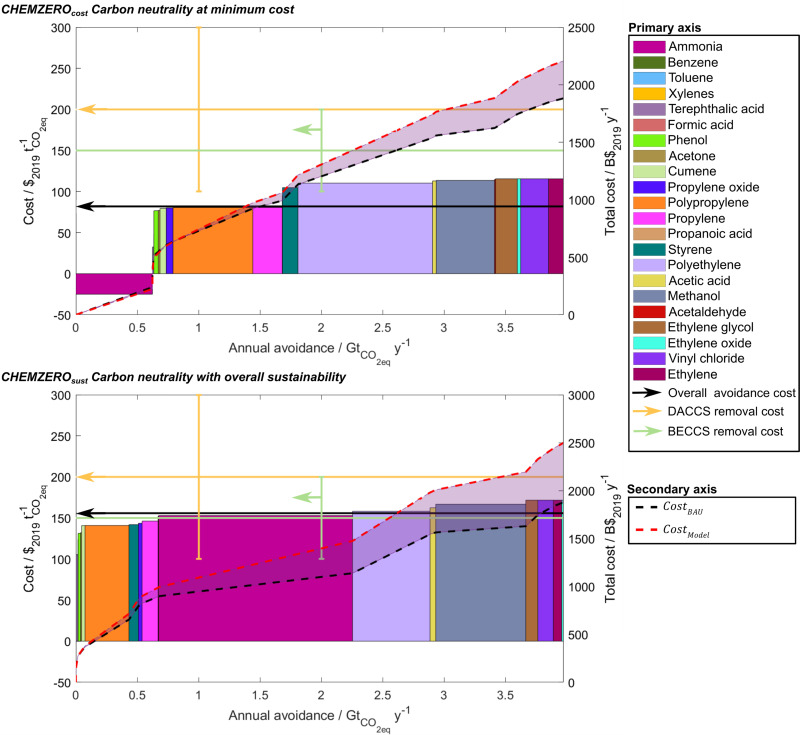
Merit order of chemicals, indicating the CO_2_ avoidance cost (primary axis) of each chemical (bars) and the total chemical system (black arrow). In addition, we also show the forecasted removal cost, rather than the avoidance cost, for DACCS and BECCS (light orange and green arrow, respectively).^57^ We further provide the total cost (secondary axis) for the respective solution (red dash line) and the *BAU* (black dashed line) to meet the 2050's annual avoidance target, equal to 4.0 Gt of CO_2eq_. The merit order depicts the sequence of the chemical's appeal in the two solutions, replacing the fossil-based pathways entirely, or partially (hybrid), with their renewable alternative to attain a carbon-neutral operation for the chemical system. The avoidance cost is calculated based on eqn (5) and (6). The transition to a carbon-neutral production of the two solutions will require a premium of 16.5 and 33.0% for the *CHEMZERO*_*cost*_ and *CHEMZERO*_*sust*_ solution, respectively, which can be translated in terms of a CO_2_ avoidance cost that ranges between 81.9–155.9 $ t_CO_2eq__^−1^. Benzene, toluene, and xylenes are not represented since the cracking process is not substituted in any of the two solutions.

We finally compare carbon mitigation *via* CCU against carbon removal *via* DACCS and BECCS, the most promising negative emissions technologies (NETs). *CHEMZERO*_*cost*_ and *CHEMZERO*_*sust*_ avoid emitting 4.0 Gt_CO_2eq__ year^−1^ in 2050, increasing the chemicals cost by 311.0–620.5 B$_2019_, respectively (Fig. 6). This amount is comparable to the 2021 Gross Domestic Product (GDP) of Finland or Belgium.^55^ Hence, both solutions would require subsidies to become competitive.

This cost is mainly linked to the high energy requirements and the levelised cost of electricity of the carbon-negative energy system covering primarily the eH_2_ needs (58.4–90.1 $ MW h^−1^, resulting in eH_2_ at 2.6–4.0 $ kg_H_2__^−1^). Moreover, the CO_2_ feedstock cost would be 62.5 $ t_CO_2__^−1^ in *CHEMZERO*_*cost*_, which is comparable to the current capture costs in coal and natural gas plants (36–53 and 48–111 $ t_CO_2__^−1^, respectively).^56^ Notably, the required premium results in an avoidance cost of 81.9 $ t_CO_2eq__^−1^, which is cheaper than the forecasted DACCS and BECCS removal costs (*i.e.*, 100.0–300.0, and 100.0–200.0 $ t_CO_2__^−1^, respectively).^57^*CHEMZERO*_*sust*_ would increase the CO_2_ feedstock and avoidance cost to 88.7$ t_CO_2__^−1^ and 155.9 $ t_CO_2eq__^−1^, respectively, and thus, still remain competitive against DACCS and BECCS. The merit order plots show that most of the avoidance is associated with chemicals with a very large demand, such as ammonia, methanol and polymers (*i.e.*, >190 Mt year^−1^). The order of the chemicals in the figure depends on the solution, where the avoidance cost of the chemicals increases when moving from *CHEMZERO*_*cost*_ to *CHEMZERO*_*sust*_. Low-carbon ammonia emerges as particularly attractive in *CHEMZERO*_*cost*_, even displaying a negative avoidance cost due to the CO_2_ feedstock it provides (*via* SR-HB coupled with CCU). In contrast, it shows a higher avoidance potential in *CHEMZERO*_*sust*_ for a significantly higher avoidance cost since it consumes a vast amount of carbon negative eH_2_.

Analysing the carbon storage capacity required by CCU *vs.* that of NETs, we find that the *BAU* directly emits (at the production sites, Fig. 4) 2.2 Gt of CO_2_ (and 4.0 Gt_CO_2eq__ on a life cycle basis). In contrast, *CHEMZERO*_*cost*_ and *CHEMZERO*_*sust*_ directly emit 1.2 and 1.3 Gt year^−1^ of CO_2_, respectively, which are essentially compensated by the carbon-negative electricity from BECCS and the DAC CO_2_, making chemicals carbon neutral from cradle-to-gate. Notably, the CCU solutions would need to store 1.63 Gt_CO_2__ year^−1^ (in *CHEMZERO*_*cost*_) and 1.56 Gt_CO_2__ year^−1^ (in *CHEMZERO*_*sust*_) from BECCS in 2050 to reach carbon neutrality. In contrast, making fossil chemicals carbon neutral from cradle-to-gate *via* CCS coupled with existing infrastructure or NETs (*e.g.*, DACCS and BECCS) would require at least 4.0 Gt_CO_2__ year^−1^ of geological storage capacity. This value corresponds to the GHG footprint of the fossil chemical system, as shown in Fig. 6.

The chemical sector has been a key driver of economic growth (*i.e.*, 7% of the world GDP in 2019),^58^ and it remains unclear how the estimated 311.0–620.5 B$_2019_ year^−1^ needed to become carbon neutral would affect its performance. Nonetheless, global inaction in climate change mitigation would also require substantial worldwide investments for climate adaptation to the 2 °C of warming, *i.e.*, 140.0–300.0 B$_2021_ year^−1^ in 2030 and 280.0–500.0 B$_2021_ year^−1^ in 2050.^59^ From a broader perspective, the UN reported that 265.0 B$_2017_ year^−1^ would be required to achieve globally SDGs 1 (no poverty) and 2 (zero hunger) by 2030.^60^ At the same time, meeting SDG 3 in 67 low- and middle-income countries (representing 95% of this group population) would need 274.0–371.0 B$_2014_ year^−1^ of additional health spending.^61^ Moreover, regarding SDG 5 (gender equality), the cost of a lower female employment rate in 2013 in the EU was estimated to be 370.0 B€_2013_ (491.0 B$_2013_),^62^ accounting for (i) resource and (ii) public finance costs. Hence, the investment for attaining carbon neutrality in the chemical sector, based on the technologies considered here, would be similar to those needed for meeting some SDGs at the global or European level. Notwithstanding this, the chemical sector should drastically reduce its fossil carbon emissions to operate sustainably, more so considering that the safe operating space should be shared among all economic sectors.

Lastly, we repeated the calculations including three additional technologies, less mature but which may play a role in the future power mix, *i.e.*, concentrated solar power, solar photovoltaics integrated with utility-scale battery storage, and wind photovoltaics integrated with utility-scale battery storage (Section S2.6 in ESI†). We found that the results would not vary substantially (≤0.2% change in cost and 2.3–12.9% in transgression, obtaining very similar portfolios). Moreover, we also conducted a sensitivity analysis on key model parameters, identifying the production cost of the chemicals, the levelised cost of electricity, and the water electrolysis efficiency as the most influential parameters, always obtaining similar solutions and insights (Section 2.4 in ESI†). Finally, using price elasticities, we investigated the extent to which the demand of chemicals would drop due to the cost increase in the carbon-neutral designs (Section 2.5 in ESI†). We found that the effect of price elasticities is low, leading to very similar demands. Besides, subsidies on low carbon chemicals would result in lower prices and lower demand changes.

## Conclusions and outlook

**Fig. 7:**
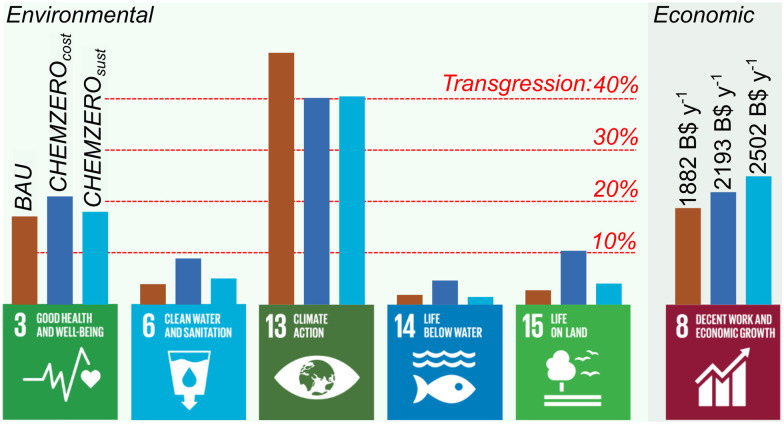
Summary of the SDGs performance of the three solutions. Bars on SDGs 3, 6, 13, 14 and 15 represent the maximum transgression across the LCA indicators within the respective SDG as depicted in Fig. 3 (*i.e.*, considering the thresholds defined for each of them), while the bar on SDG 8 depict the total cost.

Here, we assessed how CCU at a large scale could affect our ability to attain the UN SDGs. We found that the current fossil-based chemical industry could hamper the attainment of SDGs 3 and 13 due to its high impact on particulate matter and climate change (Fig. 7). CCU could help meet SDG 13 with a penalty on SDGs 3, 6, 14, and 15 due to the large energy requirements, resulting in high impacts on human toxicity, particulate matter, water scarcity, and minerals and metals depletion. The collateral damage on SDGs 6, 14 and 15 could be reduced by deploying hybrid CCU/fossil roadmaps optimised considering the SDGs. However, the high impact on SDG 3, driven mainly by toxicity and particular matter, would still be of concern, although it could be alleviated using other measures like electrostatic separations and selective non-catalytic reduction.

Moreover, CCU impacts could be further reduced by lowering the energy requirements. Mechanical and chemical recycling of polymers into monomers (or syngas) could curtail the fossil and renewable-carbon demand for chemicals and, consequently, the electrolytic hydrogen (and energy) needs.^63^ Additionally, biomass could be used as a source of chemicals, *via* syngas generation or tailored synthesis routes to, *e.g.*, biopolymers, further decreasing the carbon, hydrogen and energy requirements. Furthermore, improving power technologies like solar panels and windmills by, *e.g.*, optimising their supply chains and recycling existing and alternative construction materials, could further mitigate impacts. Lastly, optimising water consumption and product yields, possibly *via* direct electro routes with high efficiencies not relying on green methanol as an intermediate, could also further improve the environmental appeal of CCU.

From a technical viewpoint, integrating fossil technologies with CCU, DAC and BECCS seems the way forward to attain carbon neutrality with balanced economic and SDGs performance. DAC and BECCS have not been deployed at scale yet, so early adoption of these technologies will be essential to overcome technical and socio-political barriers.^64^

Attaining carbon neutrality *via* CCU will very likely require governments' financial support through subsidies (Fig. 6 and 7). However, under carbon neutrality constraints, CCU might be economically competitive with DACCS and BECCS, with the added advantage of requiring much less geological storage. Although the latter has been estimated at 2082 Gt_CO_2__,^65^ it will have to be shared among all sectors and NETs, creating intense global and regional competition for this limited resource.

Overall, our work highlights the inherent trade-offs between SDGs faced in technology development for sustainable energy and chemicals provision, which reinforces the need to embrace a range of sustainability metrics beyond the global warming impact in current assessments. In this context, LCAs based on the SDGs and PBs concepts provide a comprehensive framework to perform such evaluations and guide research and policy-making more sensibly.

## Author contributions

I. I. and G. G.-G. conceived the research. I. I., A. G.-M., J. P.-R., and G. G.-G. designed the study. I. I. developed the model formulation, carried out the analyses, and created the illustrations. I. I. and G. G.-G. wrote the paper. All authors contributed to identifying data, interpreting the results, and revising the manuscript.

## Conflicts of interest

There are no conflicts to declare.

## Supplementary Material

EE-016-D2EE01153K-s001
