# Supplementary material for: Trade-offs between Sustainable Development Goals in carbon capture and utilisation
Source: Energy Environ Sci. 2022 Aug 31;16(1):113–24. doi: 10.1039/d2ee01153k (PMC9847469; doi:10.1039/d2ee01153k)
Supplement: EE-016-D2EE01153K-s001 [file EE-016-D2EE01153K-s001.pdf]

Electronic Supplementary Material (ESI) for Energy & Environmental Science.
This journal is © The Royal Society of Chemistry 2022

Trade-offs between Sustainable Development Goals in carbon capture and utilisation

Iasonas Ioannou,[a] Ángel Galán-Martín,[b,c] Javier Pérez-Ramírez[a,*] and Gonzalo Guillén Gosálbez[a,*]

[a] *Institute for Chemical and Bioengineering, Department of Chemistry and Applied Biosciences, ETH Zürich, Vladimir-Prelog-Weg 1, 8093 Zürich, Switzerland*
[b] *Department of Chemical, Environmental and Materials Engineering, Universidad de Jaén, Campus Las Lagunillas s/n, 23071 Jaén, Spain*
[c] *Center for Advanced Studies in Earth Sciences, Energy and Environment. Universidad de Jaén, Campus Las Lagunillas s/n, 23071 Jaén, Spain*

This document contains the electronic supplementary materials for the article "Trade-offs between Sustainable Development Goals in carbon capture and utilisation". The document is organised as follows. In Section 1, the methods are explained, including the description of the $CHEMZERO$ model (mathematical formulation, data, and assumptions), while in Section 2, some additional results are presented. The last section provides the model notation.

Contents

1. Methods 2
1.1. Problem statement 2
1.2. Model description 2
Mass and energy balance constraints 7
Energy system constraints 8
Economic constraints 11
Environmental constraints 11
Objective function 12
1.3. Battery storage for solar and wind generation 13
1.4. Assumptions and limitations of the $CHEMZERO$ analysis 14
General remarks: 14
Techno-economic study: 15
LCA study: 15
Energy system modelling 16
2. Additional results 17
2.1. The power mix 17
2.2. Carbon footprint breakdown based on individual chemicals 20
2.3. The SDGs performance of the chemical system 20
2.4. Sensitivity analysis 23
Uncertain parameters and variation rate 23
Sensitivity $CHEMZEROcost$ 24
Sensitivity $CHEMZEROsust$ 35
2.5. Price elasticity of demand 38
Discussion 38
Regression of the price elasticity of demand 39
Validation based on the expected demand for methanol 39
Recalibration for the other chemicals 39
Post-optimal analysis 40
2.6. Expanding the power technologies portfolio 41
Discussion 41
Overall environmental performance 42
Chemical system design 43
Carbon-negative power to produce carbon-neutral chemicals 45
3. Model notation 46
4. References 48

1. Methods

We first formally state the problem of interest and then present the mathematical formulation to tackle it.

1.1. Problem statement

We aim to quantify the sustainability implications of the chemical sector's alignment with a carbon neutrality target in 2050 based on carbon capture and utilisation (CCU). This transition will require electrolytic hydrogen (eH_2), a vast amount of green power, and carbon dioxide (CO_2).

We consider a set of technologies to design a bespoke energy system that will aid in decarbonising 22 chemicals. Essentially, we start with a set of chemical production processes and the associated technical, economic, and environmental data. The final global demand can be met by conventional (fossil) processes or via alternative low-carbon pathways. The carbon-neutrality target is achieved by considering the chemical system as a single unified entity, to which individual chemical technologies contribute based on their inherent characteristics.

The alternative low-carbon technologies convert captured CO_2 and eH_2 via green methanol (MeOH process) into platform chemicals (methanol-to-olefins, MTO, and methanol-to-aromatics, MTA, process). We further consider three Haber-Bosch (HB) configurations for producing ammonia, e.g., (i) conventional HB starting with natural gas (NG) steam reforming (SR-HB); (ii) SR-HB with CCU (SR-HB-CCU); and (iii) eH_2 based HB (eHB). The set of generation technologies supply power to the eH_2, direct air capture (DAC), and methanol-based processes, while the global mix meets the remaining conventional production technologies' energy needs. The temporal scope corresponds to the yearly production of the chemical system.

The goal of the analysis is to determine the optimal configuration of the (integrated) energy and chemical systems to deliver the final demand of chemicals while reaching carbon-neutrality at (i) minimum cost, and (ii) at minimum transgression-based level. To identify such solutions, we developed a linear programming (LP) model, referred to as $CHEMZERO$, which we describe in detail next.

1.2. Model description

We integrate a set of chemical technologies (**Table 1**), including standard fossil technologies, CCU (green MeOH production), and olefins and aromatics production from methanol (MTO and MTA) to satisfy the demand for 22 chemicals. We further integrate the chemical technologies with a bespoke energy system comprising a range of power technologies (**Table 2** and **Table 3**). The optimal configuration is identified by solving an LP model inspired by the works by Kim et al.[1] and Ioannou et al.[2] Specifically, our formulation modifies and extends our original formulation to integrate a bespoke energy system, while covering a wide range of chemical technologies that model the bulk of the chemical sector. Furthermore, the energy system model is based on the work by Galán-Martín et al.[3] Moreover, we apply the LCIA-PBs-SDGs method developed by Sala et al.[4] to evaluate the sustainability performance of the proposed configurations.[4]

The model is based on the process network depicted in **Fig. 2** of the main article, which links the power and chemical production technologies with the respected materials (raw materials, products, and by-products) and utilities (i.e., electricity, heating, cooling, and waste treatment). We use regular letters for the parameters, while variables are written in *italics*.

We consider a set of technologies j that generate different types of products in the set I: (i) final chemical products $i \in I^{FP}$ following conventional and non-conventional pathways ($j \in CONV$ and $\in NCONV$, respectively), (ii) power $i \in I^{PP}$ –generated with power technologies $j \in PP$–, and (iii) steam and high-temperature heat $i \in I^{STEAM} \cup I^{HT}$ –produced with heat generation technologies $j \in HP$. Each technology j is described by the following parameters: (i) consumption/production rate of product i (μ_{ij}), (ii) cost (ct_j, which includes CAPEX and OPEX expenditures), and (iii) environmental impact in category $d \in D$ ($IMPT_{jd}$). Moreover, each intermediate product ($i \in I^{IP}$) has a cost (c_i), and an environmental impact d embodied in it ($IMPP_{id}$). Finally, each final chemical product $i \in I^{FP}$ has a demand (De_i) and each conventional technology with main product the chemical $i \in I^{FP}$, $j \in CONV^{FP}$, has an upper bound on its production volume (PV_j), **Table 4**.

Table 1. Chemical technologies and their sources.

Product i	Technology $j \in CONV \cup NCONV$	Data source
eH_2	Polymer electrolyte membrane (PEM) electrolysis[a]	D'Angelo et al.[5]
CO_2	Low-temperature solid sorbent DAC[b]	Fasihi et al.[6]
Methanol	Steam reforming of natural gas	Ecoinvent v3.5[7]
Methanol	Direct CO_2 hydrogenation (green MeOH)	González-Garay et al.[8]
Ethylene	Steam cracking of naphtha	Ecoinvent v3.5[7]
Ethylene	Methanol-to-olefins (MTO) process	Ioannou et al.[2]
Propylene	Steam cracking of naphtha	Ecoinvent v3.5[7]
Propylene	Methanol-to-olefins (MTO) process	Ioannou et al.[2]
Benzene	Product from catalytic reforming	Ecoinvent v3.5[7]
Benzene	Methanol-to-aromatics (MTA) process	Galán-Martín et al.[9]
Toluene	Catalytic reforming of naphtha	Ecoinvent v3.5[7]
Toluene	Methanol-to-aromatics (MTA) process	Galán-Martín et al.[9]
Xylene	Catalytic reforming of naphtha	Ecoinvent v3.5[7]

Xylene	Methanol-to-aromatics (MTA) process	Galán-Martín et al.[9]
Ammonia	Conventional HB from natural gas (SR-HB)	D'Angelo et al.[5]
Ammonia	SR-HB with CCU (SR-HB-CCU)	D'Angelo et al.[5]
Ammonia	eH_2 based HB (eHB)	D'Angelo et al.[5]
Propanoic acid	Ethylene carbonylation	Ecoinvent v3.5[7]
Ethylene oxide	Ethylene oxidation	Ecoinvent v3.5[7]
Formic acid	Hydrolysis methyl formate, via methanol carbonylation	Ecoinvent v3.5[7]
Acetaldehyde	Direct oxidation of ethylene (Hoechst-Wacker process)	Ecoinvent v3.5[7]
Acetone	Hock process	Ecoinvent v3.5[7]
Acetic acid	Celanese process	Ecoinvent v3.5[7]
Styrene	Ethylbenzene dehydrogenation, via ethylene-benzene alkylation	Ecoinvent v3.5[7]
Cumene	Alkylation of benzene and propene	Ecoinvent v3.5[7]
Phenol	Hock process	Ecoinvent v3.5[7]
Terephthalic acid	Oxidation of *p*-xylene	Ecoinvent v3.5[7]
Ethylene glycol	Oxidation of ethylene oxide	Ecoinvent v3.5[7]
Propylene oxide	Chlorohydrin process	Ecoinvent v3.5[7]
Polyethylene[c]	Mix of commercial technologies	Ecoinvent v3.5[7]
Polypropylene[c]	Mix of commercial technologies	Ecoinvent v3.5[7]
Vinyl chloride	Ethylene chlorination and oxychlorination	Ecoinvent v3.5[7]

[a] Efficiency of 80% based on the lower heating value of H_2.
[b] Based on the forecast for 2050.[6]
[c] Slurry-suspension polymerisation, bulk-suspension polymerisation, gas-phase polymerisation using Ziegler-Natta and Metallocene catalysts.[7]

Table 2. Capacity factor (CF_j) and levelised cost of electricity contributors for the various power generation technologies.

Technology $j \in PP$	CF_j (-)	Capital cost	Variable cost	Fixed O&M ($ MWh^{-1})	Transmission cost	Data source
NG power plant	0.87	7.80	1.61	31.26	1.10	EIA[10]
Coal power plant	0.85	41.95	5.48	22.75	1.08	EIA[10]
NG power plant, CCS	0.87	17.50	4.50	50.60	1.20	EIA[11]
Coal power plant, CCS	0.85	47.90	11.20	36.50	1.20	EIA[11]
NG power plant, CCU	0.87	17.50	4.50	50.60	1.20	EIA[11]
Coal power plant, CCU	0.85	47.90	11.20	36.50	1.20	EIA[11]
Bioenergy	0.83	32.32	17.38	35.84	1.15	EIA[10]
Bioenergy, CCS	0.83	56.40	44.47	57.76	1.27	EIA[11]
Wind onshore	0.41	25.96	7.46	0.00	2.57	EIA[10]
Wind offshore	0.43	58.94	29.36	0.00	2.52	EIA[10]
Nuclear	0.90	48.93	15.51	2.38	1.04	EIA[10]
Solar	0.29	20.38	6.12	0.00	3.39	EIA[10]
Hydro	0.54	39.38	11.05	3.97	2.01	EIA[10]
Geothermal	0.90	19.49	15.78	1.17	1.34	EIA[10]
Concentrated solar thermal[a]	0.61	39.01	2.89	9.03	0.00	NREL[12]
Wind | storage[a]	0.74	See **Section 1.3**				EIA[11] and
Solar PV |storage[a]	0.63	See **Section 1.3**				EIA[11] and

[a] These technologies are only used in the additional analysis presented in **Section 2.6** of the **ESI**, and are omitted in the analysis on the main manuscript.

Table 3. Activities chosen to model the electricity technologies $j \in PP$.

$j \in PP$	Activity name	Data source
NG power plant[a]	Electricity | natural gas	Ecoinvent v3.5[7]
Coal power plant[b]	Electricity |hard coal	Ecoinvent v3.5[7]
NG power plant, CCS[a]	Electricity |natural gas (Adjusted)	Ecoinvent v3.5[7] and others[13,14]
Coal power plant, CCS	Electricity | hard coal (Adjusted)	Ecoinvent v3.5[7] and others[14,15]
NG power plant, CCU[a]	Electricity |natural gas (Adjusted)	Ecoinvent v3.5[7] and others[13]
Coal power plant, CCU	Electricity |hard coal (Adjusted)	Ecoinvent v3.5[7] and others[15]
Bioenergy	Electricity | wood, future	Ecoinvent v3.5[7]
Bioenergy, CCS	Electricity | wood, future (Adjusted)	Ecoinvent v3.5[7] and others[14,16,17]
Bioenergy CHP[c]	Electricity |wood chips, 6667 kW	Ecoinvent v3.5[7]
Bioenergy CHP, CCS[c]	Electricity | wood chips, 6667 kW (Adjusted)	Ecoinvent v3.5[7] and others[14,16,17]
Wind onshore	Electricity |wind, >3MW turbine, onshore	Ecoinvent v3.5[7]
Wind offshore	Electricity | wind, 1-3MW turbine, offshore	Ecoinvent v3.5[7]
Nuclear	Electricity | nuclear, pressure water reactor	Ecoinvent v3.5[7]
Solar[d]	Electricity | photovoltaic, 3kWp facade installation	Ecoinvent v3.5[7]
Hydro	Electricity | hydro, reservoir, non-alpine region	Ecoinvent v3.5[7]
Geothermal	Electricity | deep geothermal	Ecoinvent v3.5[7]
Concentrated solar thermal[f]	Electricity | solar thermal parabolic trough	Ecoinvent v3.5[7]
Wind | storage[e,f]	Electricity |wind, >3MW turbine, onshore (Adjusted)	Ecoinvent v3.5[7] and others[18]
Solar PV |storage[e,f]	Electricity | photovoltaic, 3kWp facade installation (Adjusted)	Ecoinvent v3.5[7] and others[18]

[a] Combined cycle power plant of type 400MWe with a gas turbine of type 260 MWe and a steam turbine 140 MWe, with an efficiency between 50-60%.

[b] Hard coal power plant input is a market mix of 100MWe power plants (representing all power plants with a capacity below 250 MWe and 7% of the installed capacity) and 500 MWe power plants (representing all power plants with a capacity above 250MWe and 93% of the installed capacity). The average efficiency is considered to be ~34%

[c] Production of heat and electricity with wood chips in a state-of-the-art (in 2014) co-generation plant with a capacity of 2000 kW (referring to fuel input) in Switzerland. The total efficiency is 60%, of which 50% is thermal and 10% electric.

[d] Low voltage.

[e] See **Section 1.3**.

[f] These technologies are only used in the additional analysis presented in **Section 2.6** of the **ESI**, and are omitted in the analysis on the main manuscript.

The portfolio of heat sources $j \in HP$ comprises: (i) natural gas-based heat, (ii) heat from sources other than natural gas, (iii) steam directly from natural gas, and (iv) steam from CHP power plants, with the activities and prices being extracted from the Ecoinvent v3.5 database.[7] We further classify the electricity generation technologies $j \in PP$ into (i) dispatchable, $j \in PP^{DIS}$, (e.g., coal with/without CCS, natural gas with/without CCS, bioenergy with/without CCS, nuclear, hydropower reservoir, geothermal power plants, concentrated solar power [CSP], and onshore wind and solar PV with storage), and (ii) non-dispatchable, $j \in PP^{NDIS}$, (e.g., solar PV, and onshore/offshore wind) sources. Please note that CSP, wind onshore and solar PV with storage are only used in the additional analysis presented in **Section 2.6** of the **ESI**, and are omitted in the analysis on the main manuscript. Finally, we classify the energy sources $s \in S$ of the power technologies ($j \in PP$) into 7 categories, i.e., s, (i) coal, (ii) natural gas, (iii) biomass, (iv) wind, (v) hydropower, (vi) solar, and (vii) geothermal, $j \in PP^s$.

The LP finds the optimal production rates of chemicals and energy generation within the system (continuous variable W_j), and the amount of purchased products (continuous variable P_i), considering the constraints described below.

Mass and energy balance constraints

The mass and energy balances must be satisfied for each product i, i.e., material ($i \in I^{FP} \cup I^{IP}$), heat ($i \in I^{STEAM} \cup I^{HT}$), and power ($i \in I^{PP}$), as given in **Eq. 1**, where the amount of product purchased, P_i, plus the amount produced equals the amount consumed plus the sales, S_i:

$$P_i + \sum_{j \in OUT(i)} \mu_{ij} W_j = \sum_{j \in IN(i)} \mu_{ij} W_j + S_i, \ \forall i \tag{1}$$

Furthermore, we enforce that the sales of final products should equal the final demand (De_i), given in **Eq. 2** and **Table 4**, while the only end-user of the generated energy and intermediate products is the chemical system (**Eq. 3**).

$$S_i \geq De_i,\ \forall i \in I^{FP} \tag{2}$$

$$S_i = 0,\ \forall i \in I^{STEAM} \cup I^{HT} \cup I^{PP} \cup I^{IP} \tag{3}$$

Finally, we avoid expanding in capacity the fossil technologies that deliver as main product the final chemical product i, i.e., $i \in I^{FP}, j \in CONV^{IP}$**,** by forcing their annual production volume (PV_j [**Table 4**]) to lie below the one predicted for 2050.

$$W_j \leq PV_j,\ \forall j \in CONV^{FP} \tag{4}$$

Energy system constraints

Technologies $j \in PP$ generate power, and their production rate (W_j) is divided into standard and backup (Gen_j^{ST} and Gen_j^{BACKUP}) generation types.

$$Gen_j^{ST} + Gen_j^{BACKUP} = W_j,\ \forall j \in PP \tag{5}$$

The total power (TP) is given by the summation of the generated amount of each technology $j \in PP$.

$$TP = \sum_{j \in PP} W_j \tag{6}$$

The installed capacity can then be calculated via the CF_j of the respective technology j and the annual operational hours (H, e.g., 8760 h). The CF_j is the ratio of the power output generated within a year over the maximum possible output (**Table 2**).

$$Gen_j^{BACKUP} = CAP_j^{BACKUP} \cdot CF_j \cdot H,\ \forall j \in PP^{DIS} \tag{7}$$

$$Gen_j^{ST} \leq CAP_j^{ST} \cdot CF_j \cdot H,\ \forall j \in PP \tag{8}$$

Notably, due to the firmness of the standard sources, their installed capacity is constrained by an inequality. The generated electricity from backup technologies ensures the system's reliability. Thus, **Eq. 7** is expressed as an equality constraint. The following equation is then formulated to ensure a reliable energy system:

$$\sum_{j \in PP^{DIS}} CAP_j^{BACKUP} = BACKUP \sum_{j \in PP^{NDIS}} CAP_j^{ST} \tag{9}$$

The parameter $BACKUP$ was selected to be equal to 0.5. The physical interpretation of this constraint is that each MW installed of an intermittent renewable technology requires installing an additional 0.5 MW of firm technologies to hedge supply in periods with unfavourable weather conditions. Notably, non-dispatchable technologies, due to their seasonal and daily variability, cannot generate backup power.

$$CAP^{BACKUP}_{j} = 0,\ \forall j \in PP^{NDIS} \tag{10}$$

Furthermore, we need to ensure that the electricity needs of other sectors can be satisfied, considering the availability of resources. Thus, we limit the generated power for the chemical system according to the global technical potential ($Tech^{Pot}_{s}$),[19] while considering the amount already consumed by other anthropogenic activities (Mix^{2050}_{s})[20] (**Table 5**). In **Eq. 11**, a_{js} links technologies $j \in PP$ with the corresponding power source s.

$$\sum_{j \in PP^{S}} W_j \cdot a_{js} \le \left(Tech^{Pot}_{s} - Mix^{2050}_{s}\right),\ \forall s \in S \tag{11}$$

Finally, we introduce a maximum annual growth rate parameter ($CAAGR_s$) for power technologies, defined based on sensible assumptions.[20,21] The bounds for the different technologies are provided in **Table 5**. Essentially, this inequality constraint (**Eq. 12**) limits the technology's development. The starting period for installing power technologies is 2021. Thus, the availability of power generation considers the given time horizon, e.g., from 2021 – 2050, and the amount generated in the starting year (Mix^{2021}_{s}).

$$\sum_{j \in PP^{S}} W_j a_{js} \le Mix^{2021}_{s}\left(1 + CAAGR_s\right)^{(2050-2021)} - Mix^{2050}_{s},\ \forall s \in S \tag{12}$$

Table 4. Production volume of the BAU system and final demand of product $i \in I^{FP}$.

Product $i \in I^{FP}$	Production volume[a] (Mt)	Final demand (Mt)	Reference year	Data source
Methanol	213.5	196.9	2050	IEA[22]
Ethylene	455.4	31.7	2050	IEA[22]
Propylene	291.7	70.8	2050	IEA[22]
Benzene	156.5	20.4	2050	IEA[22]
Toluene	71.2	71.2	2050	IEA[22]
Xylene	163.7	65.2	2050	IEA[22]
Ammonia	533.7	533.7	2050	IEA[22]
Propanoic acid	1.0	1.0	2013[c]	Market data[23]
Ethylene oxide	64.0	8.5	2050	IEA[22]
Formic acid	2.4	2.4	2016[c]	Aresta et al.[24]
Acetaldehyde	3.2	3.2	2015[c]	Market data[25]
Acetone	17.6	17.6	2050	See[b] and IEA[22]
Acetic acid	30.5	23.0	2022[c]	Market data[26]
Styrene	106.7	106.7	2050	IEA[22]
Cumene	78.3	39.9	2050	IEA[22]
Phenol	28.5	28.5	2050	IEA[22]
Terephthalic acid	149.4	149.4	2050	IEA[22]
Ethylene glycol	78.3	78.3	2050	IEA[22]
Propylene oxide	21.3	21.3	2050	IEA[22]
Polyethylene	270.4	270.4	2050	IEA[22]
Polypropylene	177.9	177.9	2050	IEA[22]
Vinyl chloride	135.2	135.2	2050	IEA[22]

[a] Based on the BAU system.
[b] Based on the phenol production, as a co-product.
[c] Updated to 2050, with the same assumptions used in the IEA[22] for the other chemicals.

Economic constraints

The total cost (TC) of $CHEMZERO$ is given by the technology's costs plus the purchases of

$$TC = \sum_{j \in J} W_j \cdot ct_j + \sum_{i \in I^{IP}} P_i \cdot c_i \qquad (13)$$

intermediate products as shown in **Eq. 13**:

Table 5. Bounds used for the technical potential of the power technologies.

s	Coal	Natural gas	Nuclear	Hydro	Bio	Wind	Geo-thermal	Solar PV	CSP[b]
$Tech^{\text{[illegible]}}_s$ (PWh)	10.1	12.2	5.4	13.9[19]	8.3[27]	23.6[19]	32.8[19]	437.5[19]	See[c]
$CAAGR_s$ (%)[a]	5.0	5.0	2.7	2.8	6.6	7.9	8.0	9.9	17.5
$Mix^{\text{[illegible]}}_s$ (PWh)	9.0	6.5	2.8	4.5	0.8	1.8	0.1	1.0	2.1 $\times 10^{-2}$
$Mix^{\text{[illegible]}}_s$ (PWh)[a]	0.9	2.4	4.3	8.1	3.5	11.6	0.8	11.4	1.6

[a] The world energy outlook provides data for 2018-2040.[20] Thus, values for $Mix^{\text{[illegible]}}_s$ are based on calculations applying sensible assumptions based on the trends observed in 2018-2040. Similarly, the Mix^{2021}_s, is based on the trend between 2018 and 2025.
[b] CSP is only used in the additional analysis presented in **Section 2.6** of the **ESI**, and is omitted in the analysis on the main manuscript.
[b] Solar and CSP share the same technical potential.

Environmental constraints

The total impact on each category d ($TIMP_d$) corresponds to the summation of the impact embodied in the intermediate products purchased (used as feedstock) plus the impact of the technologies themselves:

$$TIMP_d = \sum_{j \in J} W_j \cdot IMPT_{jd} + \sum_{i \in I^{IP}} P_i \cdot IMPP_{id}, \ \forall d \qquad (14)$$

The transgression of each impact category d is determined in **Eq. 15**. The carrying capacities, SOS_d, of each environmental impact category d are provided in

Table 6.

$$TR_d = \frac{TIMP_d}{SOS_d},\ \forall d \in D \tag{15}$$

$CHEMZERO$ ensures a carbon-neutral operation, e.g., global warming potential impact (GW) equals zero.

$$TIMP_{d\,=\,GW} = 0 \tag{16}$$

Finally, we define non-negativity constraints on some continuous variables, i.e., production rate, products purchases and sales, and total generated power:

$$W_j, P_i, S_i, TP \geq 0 \tag{17}$$

Objective function

$CHEMZERO_{cost}$ aims to minimise the system's TC subject to the carbon neutrality target and various technical constraints (**Eq. 18**). This strategy reflects current policies and describes an emergency program to make the chemical industry carbon-neutral by the first half of the 21st century:

$$\begin{matrix} \min\{TC\} \\ s.t. \\ Eq.\,1-17 \end{matrix} \quad (CHEMZERO_{cost}) \tag{18}$$

$CHEMZERO_{sust}$ aims to minimise a single environmental metric, defined as the average transgression of the carrying capacities, subject to the carbon neutrality target and other constraints, as mentioned earlier.

$$\begin{matrix} \min\left\{SUST_{index} = \frac{\sum_d TR_d}{|D|}\right\} \\ s.t. \\ Eq.\,1-17 \end{matrix} \quad (CHEMZERO_{sust}) \tag{19}$$

Because we aggregate metrics,[28] the model attempts to minimise those categories with a higher criticality level, e.g., high transgression shares. $CHEMZERO$ may be degenerate, meaning that it may contain feasible solutions with better cost and the same sustainability level, or vice versa, as its optimal solution. Hence, a post-process step is applied to minimise the cost subject to not worsening the best sustainability performance identified in the first model run, or vice versa.

Note that the aggregated value of $SUST_{index}$ has no specific significance anymore in terms of the transgression of individual planetary boundaries.[28]

Table 6. Quality level of the LCA indicators of the EF method and carrying capacities (SOS_d) of the LCIA-PBs-SDGs.[4]

Environmental area of protection d	Quality level[29,30]	SOS_d value
Climate change (kg CO_{2eq})	I	$6.8x10^{12}$
Ozone depletion (kg $CFC11_{eq}$)	I	$5.4 x10^{8}$
Ionising radiation, HH[a] (kBq $U\text{-}235_{eq}$)	II	$7.4x10^{14}$
Photochemical ozone formation, HH (kg $NMVOC_{eq}$)	II	$4.1x10^{2}$
Respiratory inorganics[a] (disease inc.)	I	$7.3x10^{5}$
Non-cancer human health effects[a] (CTUh)	II/III	$5.8x10^{6}$
Cancer human health effects[a] (CTUh)	II/III	$1.4x10^{6}$
Acidification terrestrial and freshwater (mol H_{+eq})	II	$1.0x10^{12}$
Eutrophication freshwater (kg P_{eq})	II	$5.8x10^{9}$
Eutrophication marine (kg N_{eq})	II	$2.0x10^{11}$
Eutrophication terrestrial (mol N_{eq})	II	$6.1x10^{12}$
Ecotoxicity freshwater (CTUe)	II/III	$1.3x10^{14}$
Land use, soil erosion (kg soil loss)	III	$1.3x10^{13}$
Water scarcity (m^3 deprived)	III	$1.8x10^{14}$
Resource use, energy carriers (MJ)	III	$2.2x10^{14}$
Resource use, mineral and metals ($kgSb_{eq}$)	III	$2.2x10^{8}$

[a] Updated for 2050 based on an estimated global population of 9.72x10.[9]

1.3. Battery storage for solar and wind generation

For the expenditures of utility-scale battery storage, we use forecasted data for 2050 available in the Open Energy Data Initiative (OEDI) of the National Renewable Energy Laboratory (NREL) of the US.[12] These costs include the capital expenses (CAPEX), fixed operation and maintenance (FOM), and variable operation and maintenance (VOM) costs. In particular, here we consider a Lithium-ion battery with an 8 h duration discharge and follow the NREL assumption of approximately one cycle per day. Based on NREL's moderate scenario for 2050, the CAPEX and FOM costs are 995 \$ kW^{-1} and 25 \$ kW^{-1} y^{-1}, respectively, while no VOM costs were assumed.

The deployment of utility-scale battery storage allows increasing the capacity factor of solar and wind power. The additional cost for storage is calculated as follows:

$$Cost\ of\ storage\left[=\frac{\$}{MWh}\right]=\frac{PFF\cdot CRF\cdot CAPEX+FOM}{8760\cdot CF_{battery}}+VOM \qquad (20)$$

, where CRF is the capital recovery factor (equal to 1.022), PFF the constriction finance factor (equal to 0.043), and $CF_{battery}$ is the capacity factor of the battery $\left({}^{8}/_{24}\right)$.

Using the above mentioned parameters, the LCOE of solar and wind power generation integrated with a utility-scale battery would include an additional cost of storage equal to 23.5 \$ MWh^{-1}. Furthermore, based on the assumption of one discharge cycle per day, the availability of solar or wind power (when integrated with battery storage) increases by 8 h d^{-1}, leading to a capacity factor of 63 and 74%, respectively. Finally, based on these data, the latter integrated power technologies are considered as dispatchable sources in the $CHEMZERO$ model.

1.4. Assumptions and limitations of the $CHEMZERO$ analysis

General remarks:

- In the main manuscript, we assume an 80% efficiency for the water-splitting process (based on the lower heating value [LHV]), regarded as an optimistic value based on the range of long-term forecasts on technology improvements.[31] Besides, the electrical efficiency (LHV, %) of the (i) alkaline electrolyser, (ii) proton exchange membrane electrolyser, and (iii) solid oxide electrolysis cells, was estimated within the following range: 63-70%, 67-74%, and 77-90%, respectively. The influence of the electrolyser efficiency on the system performance was evaluated in a sensitivity analysis, as described in **Section 2.4** of the **ESI**.
- The PBs should be fairly and equitably distributed among all economic sectors, e.g., sectorial scale. This would require applying top-down allocation approaches, i.e., downscaling principles,[32] to assign shares of the total ecological budget to each anthropogenic activity. However, at the current state, there is no universally agreed downscaling approach.[33,34] Hence, we provide our results relative to the full safe operating space, avoiding the application of downscaling principles.
- The useful thermal energy from the CHP power plants is provided in the form of steam, which is available for the production facilities. Besides, CHP plants are commonly used in ammonia and fertiliser manufacturing as well as in other chemical plants, among other industrial applications.[35] We assume that the power and steam generated by the CHP plants are fully consumed internally, i.e., in the chemical system, to avoid uncertainties related to selling or wasting useful thermal energy.
- We assume that the heat recovered from the purge streams of MeOH plants replaces heat from natural gas. Again, the generated heat must be consumed within the network. Otherwise, such technologies will not be selected. Moreover, even though we envision well-designed chemical clusters, we assume 20% heat losses with the on-

site integration. Such losses may originate from the steam's distribution line or leaks from valves, pipes, and steam traps.

- We assume that the capacity of geological formations for the storage of CO_2 will not be a limiting factor. Furthermore, we assume the deployment of BECCS to the extent needed, which requires an annual storage capacity of 1.6 $GtCO_2$ according to the calculations of the main manuscript. BECCS appears in many IAMs scenarios,[36] although its large-scale implementation is not exempt from controversy.[37,38]
- In the case study of the main manuscript, we assume that the DAC's utility consumption and cost will be reduced via learning curves, based on Fasihi et al.[6] Since the development of DAC technologies is uncertain, we carry out a sensitivity analysis on the energy requirements, as described in **Section 2.4** of the **ESI**.
- In the main manuscript, we omit energy and eH_2 storage, as we consider that the bespoke mixes are fully reliable by design (**Eq. 9**). Hence, they can ensure the continuous operation of the plants by design. To provide further insight, we ran again the model considering utility-scale battery storage for the fluctuating power generation technologies (as described in **Section 1.3**) and concentrated solar power. Therefore, **Section 2.6** provides the results generated considering an expanded portfolio, including the above mentioned technologies.
- We limit the scope of this work to high technology readiness level (TRL) technologies. Nonetheless, $CHEMZERO$ could be expanded to include other novel chemical pathways within the network, which might lead to other solutions. For example, emerging plastic recycling technologies[20] are omitted in our assessment due to their low TRLs. Recycling practices would reduce the annual production volume of platform chemicals while decreasing the need for eH_2.

Techno-economic study:

- In the main manuscript, the price of the BAU chemical products is calculated based on data from the United Nations Commodity Trade Statistics Database, taking 2018 as reference year.[39] In **Section 2.4** of the **ESI,** we conduct a sensitivity analysis on the technologies cost to evaluate their influence on the overall performance.
- Moreover, we conduct a sensitivity analysis on the cost of eH_2, and the DAC process to evaluate the influence of the latter parameters on the overall performance of the system, as described in **Section 2.4** of the **ESI**.

LCA study:

- Some impact metrics (LCI entries and associated characterisation factors) are affected by more pronounced uncertainties. According to the International Reference Life Cycle Data System (ILCD) levels, LCA metrics can be classified based on their quality into three levels: —I (recommended and satisfactory), —II (recommended but in need of some improvements) or —III (recommended, but to be applied with caution). A detailed description of these levels is provided in the original source,[30] while we display in **Table 6** the quality level of the LCA indicators of the EF method. Moreover, Sala and co-workers defined a fixed zone of

uncertainty for the carrying capacities used in their LCIA-PBs-SDGs method. Accordingly, the SOS is defined as the area within the carrying capacity, the zone of uncertainty lies between one time the carrying capacity and twice its value, while the high risk zone corresponds to impacts beyond twice the carrying capacity.

- We define a cradle-to-gate scope, assuming that the BAU and alternative low-carbon routes will involve the same downstream processing and transformation of the chemical system, i.e., its conversion and further processing into other products. Carbon-neutrality, therefore, refers to a cradle-to-gate scope. However, the CO_2 would be re-emitted at the end of the chemical's lifetime if they are incinerated or disposed of in a landfill without any CO_2 capture process, or naturally degrade.
- CHEMZERO assumes a fixed final demand for the 22 chemicals, as listed in **Table 4**. Notably, the higher cost of CO_2-based chemicals could decrease the final demand based on the price elasticity. Nonetheless, such an assumption is necessary to carry out the LCA according to the ISO standards, as otherwise a different functional unit (demand) would emerge depending on the scenario (due to differences in production costs according to the pathways chosen). According to the ISO 14040, *Comparability of LCA results is particularly critical when different systems are being assessed to ensure that such comparisons are made on a common basis*. To address this issue, we evaluate the validity of the constant final demand assumption by recalculating the new demand that would result from the new prices, as explained in **Section 2.5** of the **ESI**.
- The carrying capacities (SOS_d) of the LCIA-PBs method were updated for 2050 for four LCA indicators, i.e., ionising radiation, respiratory inorganics, and non-cancer and cancer human toxicity. Notably, we assume a global population increase between 2018-2050 equal to 0.77% based on the trend of the world energy outlook for the periods 2018-2030 and 2018-2040 (annual global population increase of 0.95 and 0.86%, respectively).[20]
- We included the impact of the electrolysers in the LCA, yet no technological improvements within 2021-2050 were considered. This assumption is based on the observation that the impact embodied in the electrolyser tends to be low compared to the impact of the amount of energy needed for water splitting.[40]
- The LCI data for the construction of the MeOH, MTO, MTA, and ammonia plants are based on the methanol and organic plant activities available in Ecoinvent v3.5. The latter activity is based on an average calculated based on the BASF site of Ludwigshafen and a chemical factory in Gendorf, with an annual capacity of 50000 t, and lifetime of 50 yr, leading to 4.0×10^{-10} units per kg of produced chemical.[7]
- The cooling water evaporation losses are calculated as a fraction of the total flow following the same assumptions as in Ecoinvent v3.5,[7] and validated with an additional source.[41]

Energy system modelling

- The energy system powers the eH_2, DAC, and methanol-based processes, while the fossil-based technologies are assumed to be powered by a global mix. The reason for

this assumption is that global energy systems do not consider the energy demand of a large-scale transition to low-carbon chemicals.[20]

- We used learning curves for the cost and capacity factor of power technologies as described in the EIA reports.[10,11] However, we neglect learning curves in the environmental assessment due to the lack of life cycle inventories, while we assume that climate change will not affect significantly the average capacity factors of solar and wind power generation.[19] To study the impact of uncertainties affecting such learning curves as well as other parameters, we carry out a sensitivity analysis on the power system parameters, as described in **Section 2.4** of the **ESI**.
- For coal, natural gas, and nuclear power, we assume that their technical potential cannot exceed twice their generated power in 2018. This assumption was made to avoid the unrealistic high expansion of fossil and nuclear power facilities.
- The world energy outlook provides the generated power and the deployment rate data between 2018-2040 to satisfy the total anthropogenic needs.[20] We consider values based on sensible assumptions for the missing data according to the trends observed in the latter period. In particular, the $CAAGR_s$ values are considered to be higher than those calculated based on similar assumptions as in the world energy outlook for 2021-2050 (i.e., 1.25% higher for all technologies, except for hydropower, for which we considered a 0.75% increase since the deployment of new pumped-hydro plants will not improve further its economic performance and is expected to slow down in the upcoming years).

2. Additional results

This section contains additional results omitted in the main manuscript due to space limitations. **Table 7** and **Figure S1** provide specific information related to the energy system of the $CHEMZERO$ solutions, whereas **Figure S2** depicts the contribution of individual chemicals towards the total carbon footprint of the chemical system. Finally, we discuss further the performance of the $CHEMZERO$ model concerning the 5 SDGs and the 16 LCA indicators based on the figures of the main manuscript.

2.1. The power mix

Analysing the impact of the power mix expressed per unit of kWh produced, we find that $CHEMZERO_{sust}$ improves 12 out of the 16 LCA indicators compared to $CHEMZERO_{cost}$ (**Table 7**). Ionising radiation and resources depletion –energy– worsen substantially (62.0 and 28.5%, respectively), followed by water scarcity and ozone depletion (5.1 and 1.4%, respectively). Nonetheless, these adverse effects are outweighed (to some extent) by a 9.2% drop in power needs between the two configurations (**Fig. 5**). Focusing on the contribution of each technology toward the total impact of electricity (**Figure S1**), nuclear power emerges as the primary driver of ionising radiation, ozone depletion, and energy resource depletion. Hydropower dominates the impact in water scarcity, whereas the remaining LCA indicators are strongly linked to bio-, wind-, and solar-based power generation. The carbon footprint of $CHEMZERO_{cost}$ and $CHEMZERO_{sust}$ energy systems show similar breakdown (**Figure S1**).

Besides, BECCS provide all the carbon-negative power while $CHEMZERO_{sust}$ improvements are attained by avoiding solar, and shifting from onshore to offshore wind.

Table 7 Environmental burden of the generated power per kWh for the 16 LCA indicators of the EF method.

SDG	LCA indicator	$CHEMZERO_{cost}$	$CHEMZERO_{sust}$	Improvement
3	Human toxicity, cancer (CTUh)	$2.6x10^{-9}$	$2.2x10^{-9}$	16.2%
	Human toxicity, non-cancer (CTUh)	$5.7x10^{-8}$	$4.9x10^{-8}$	15.3%
	Particulate matter (disease inc.)	$4.6x10^{-9}$	$3.2x10^{-9}$	31.5%
	Photochemical ozone formation (kg $NMVOC_{eq}$)	$3.4x10^{-4}$	$2.6x10^{-4}$	23.6%
	Ionising radiation (kBq U-235_{eq})	$1.5x10^{-2}$	$2.4x10^{-2}$	–62.0%
6	Water use (m^3 depriv.)	$2.7x10^{-1}$	$2.8x10^{-1}$	–5.1%
	Ecotoxicity, freshwater (CTUe)	$9.8x10^{-2}$	$6.6x10^{-2}$	32.5%
13	Climate change ($kgCO_{2eq}$)	$–1.2x10^{-1}$	$–1.5x10^{-1}$	25.7%
	Resource use, energy (MJ)	1.4	1.7	-28.5%
	Ozone depletion	$8.3x10^{-9}$	$8.4x10^{-9}$	–1.4%
14	Eutrophication, marine (kg N_{eq})	$1.2x10^{-4}$	$1.0x10^{-4}$	16.0%
	Eutrophication, freshwater (kg P_{eq})	$1.4x10^{-5}$	$2.4x10^{-6}$	82.7%
15	Land use, soil erosion (kg soil loss)	$3.3x10^{-3}$	$2.3x10^{-3}$	31.6%
	Eutrophication, terrestrial (mol N_{eq})	$1.8x10^{-3}$	$1.6x10^{-3}$	11.0%
	Acidification terrestrial and freshwater (mol H_{+eq})	$4.5x10^{-4}$	$2.5x10^{-4}$	44.2%
	Resource use, mineral and metals (kg Sb_{eq})	$1.3x10^{-6}$	$2.3x10^{-7}$	82.9%

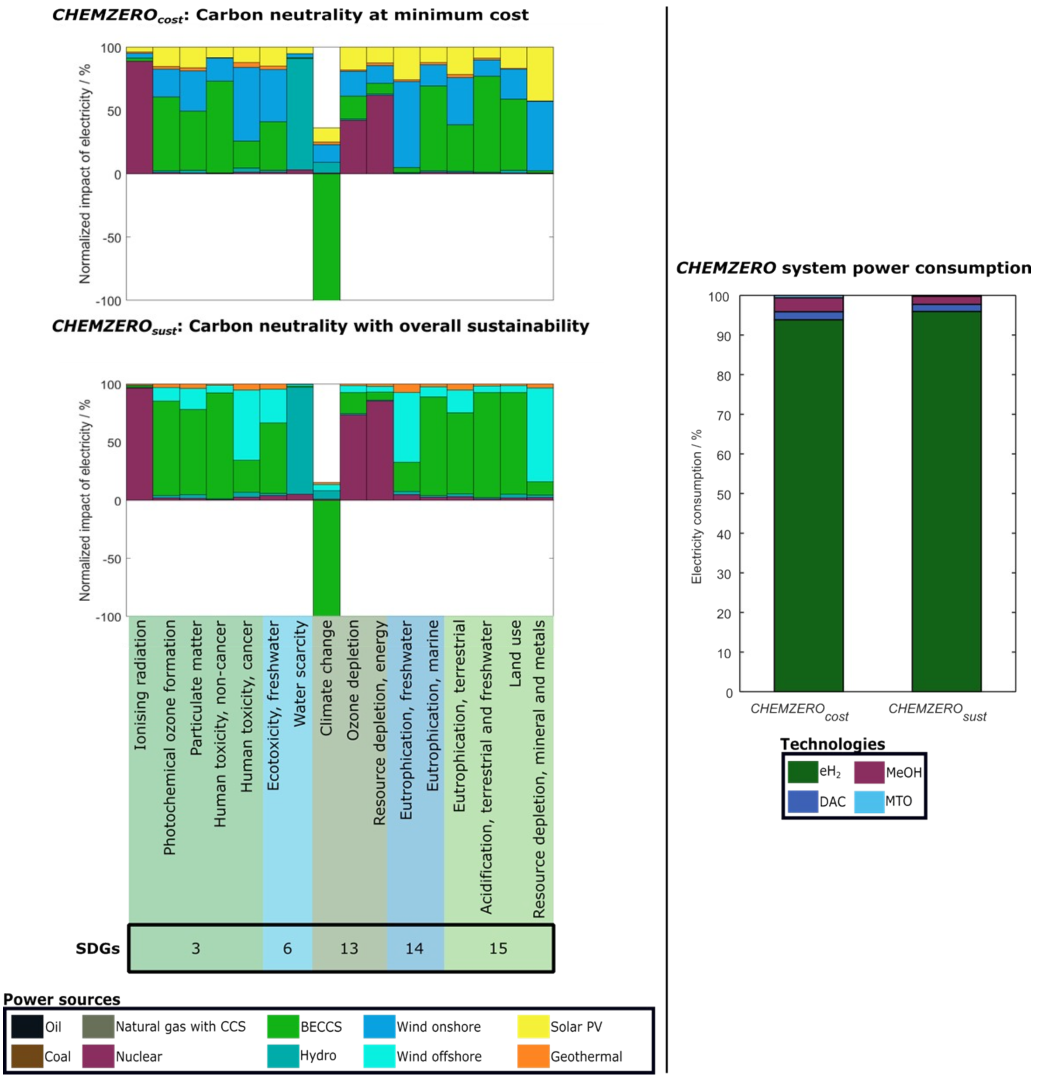

Figure S1 Breakdown of the normalised impact of the bespoke electricity mixes of the two models in the 16 LCA indicators of the EF method (left), and power consumption from the bespoke power mix for the two $CHEMZERO$ designs (right).

2.2. Carbon footprint breakdown based on individual chemicals

Focusing on the impact of individual chemicals on the carbon footprint (**Figure S2**), we observe that the carbon-neutral configurations attain reduction benefits from different chemicals. In $CHEMZERO_{cost}$, the decarbonised backbone of the chemical system, e.g., ethylene, propylene, and methanol, indirectly affects the performance of the large-volume polymers (polyethylene and polypropylene), which attain a negative carbon footprint (on a cradle-to-gate basis). Notably, the embodied impact of ammonia contributes significantly towards the total burden on this category. In contrast, $CHEMZERO_{sust}$ reduces the global warming of the system predominantly by generating methanol and ammonia with negative carbon eH_2 and using DAC CO_2. The latter leads to a configuration where the organic substances of the chemical system have higher shares of impacts when compared with the $CHEMZERO_{cost}$ configuration.

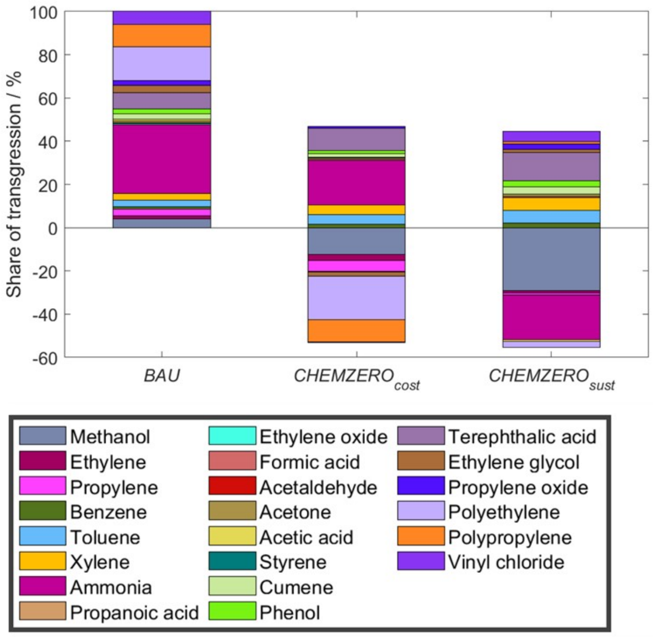

Figure S2 Breakdown of the normalised impact of the chemical system in the climate change LCA indicator of the EF method.

2.3. The SDGs performance of the chemical system

Good health and well-being goal (SDG 3)

Based on **Fig. 3** of the manuscript, the BAU heavily stresses particulate matter (i.e., 17.2% of the SOS), while performing much better in the remaining SDG 3 metrics (3.1, 2.5, 3.6, and 1.2×10^{-2}% of the boundary in human toxicity [non-cancer and cancer], photochemical ozone formation, and ionising radiation, respectively).

The $CHEMZERO_{cost}$ solution worsens all the impact categories (4.9-, 1.8-, 1.1-, 1.2-, and 2.8-fold in human toxicity [non-cancer and cancer], photochemical ozone formation, particulate

matter, and ionising radiation, respectively), leading to severe collateral damage in non-cancer human toxicity (15.1% of the SOS) and the already critical particulate matter (21.1%).

$CHEMZERO_{sust}$ outperforms $CHEMZERO_{cost}$ in 4 out of 5 SDG 3 indicators, but not the BAU (the impact worsens 3.8- and 1.5-, 9.9x10^{-1}, 1.0-, and -3.4-fold [following the same sequence as before] relative to the BAU), while slightly improving photochemical ozone formation compared to the BAU.

Focusing on the breakdown of impacts, the direct release of particulates –below 2 μm– and NO_x from the chemical production processes of the BAU, and the related emissions embodied in the grid electricity consumed are responsible for 25.8, 5.3, and 6.0% of the particulate matter impact, respectively (**Fig. 3**, –bottom–). The remaining particulate matter burden is linked to the extraction and use of fossil resources, and other direct emissions and activities. The impact on the remaining SDG 3-related categories is mostly linked to the grid electricity and other activities. $CHEMZERO_{cost}$ worsens all the SDG 3-related LCA indicators mostly due to the vast power consumption (**Fig. 3**, –bottom–, links with the power mix discussed in **Figure S1**). Direct NO_x emissions, affecting categories photochemical ozone formation, drop in the carbon-neutral solutions because they make less use of cracking-based production and steam methane reforming.

Clean water and sanitation goal (SDG 6)

The BAU solution consumes 4.0 and 1.6% of the SOS in water scarcity and freshwater ecotoxicity, respectively. The cost-effective carbon-neutral solution ($CHEMZERO_{cost}$) worsens both indicators, whereas $CHEMZERO_{sust}$ affects the two indicators only marginally (2.3- vs. 1.3- and 1.5- vs. 9.8x10^{-1}-fold increase, respectively, compared to the BAU).

In the BAU system, water scarcity is mostly linked to the evaporation losses from the cooling towers. The $CHEMZERO_{cost}$ solution worsens this indicator due to the additional MeOH plants and the hydropower facilities (**Fig. 3-4**, and **Figure S1**). In $CHEMZERO_{sust}$ this effect is partially offset by the lower MeOH throughput, compared to $CHEMZERO_{cost}$, and the deployment of eHB, which both lead to lower cooling water and power requirements. The increase in ecotoxicity freshwater in both carbon-zero solutions is attributed to the high energy needs (**Fig. 3**, –bottom–), where the superior performance of $CHEMZERO_{sust}$ is due its lower energy consumption, the shift from onshore to offshore wind turbines, and the avoidance of solar panels. Overall, the BAU solution marginally impacts SDG 6, while cost-effective decarbonisation pathways could hinder freshwater use, especially in regions with water supply shortages.

Climate action goal (SDG 13)

The BAU solution performs very poorly in the SDG13 metrics, mainly in climate change, and resource depletion –energy– (58.4 and 49.1% of the SOS, respectively), due to its strong reliance on fossil carbon. In contrast, the ozone depletion transgression level is negligible (2.9×10^{-2}%).

The BAU net direct CO_2 emissions are responsible for 56.4% of the total climate change transgression, followed by other activities, grid electricity, and heat from NG (i.e., 28.3, 10.4 and 4.9%, respectively). In contrast, other activities over the life cycle are predominantly responsible for the depletion of the resource –energy (96.1%). To achieve carbon neutrality (on a cradle-to-gate basis), the model reduces the use of fossil feedstock, decreasing resource depletion –energy– (from 49.1% to 40.3% and 40.6%, in the $CHEMZERO_{cost}$ and $CHEMZERO_{sust}$, respectively), utilises carbon negative electricity and consumes DAC CO_2. On the downside, the carbon-neutral solutions exacerbate ozone depletion (1.7- and 1.2-fold higher than the BAU), mainly due to extensive power consumption, yet their transgression levels in this category are negligible.

Life below water goal (SDG 14)

The BAU solution consumes 1.9 and 1.3% of the freshwater and marine eutrophication SOS, respectively. Both metrics worsen in the climate-neutral solutions, substantially in $CHEMZERO_{cost}$ and less critically in $CHEMZERO_{sust}$ (4.7 and 1.8% vs. 2.1 and 1.5%, respectively).

In the BAU, the direct NO_x emissions and use of grid electricity are responsible for 62 and 12.8% of the total marine eutrophication impact, respectively (**Fig. 3**, –bottom–). Moreover, electricity consumption from the bespoke mix primarily drives the impact in the carbon-neutral solutions. This is explained by the strong reliance on BECCS (**Figure S1**). The $CHEMZERO_{sust}$ solution outperforms $CHEMZERO_{cost}$ due to its lower energy consumption and its power mix composition (i.e., offshore vs. onshore, and phase-out of solar power). Furthermore, lower direct NO_x emissions in both solutions lead to better performance in marine eutrophication.

Life on land goal (SDG 15)

The BAU solution performs reasonably well in these indicators (<3% of the SOS in resource depletion –mineral and metals–, acidification, terrestrial eutrophication, and land use –soil erosion–, respectively). The alternative solutions worsen some of them substantially (10.5, 2.0, 1.0, and 0.5% vs. 4.1, 1.6, 0.8, and 0.3%, in the $CHEMZERO_{cost}$and $CHEMZERO_{sust}$, respectively, [following the same sequence as before]), less so the $CHEMZERO_{sust}$ solution that even outperforms the BAU in the acidification category.

The BAU system's breakdown reveals that different processes are responsible for the individual SDG 15-related indicators. Resource depletion –minerals and metals– is mainly affected by other activities, while land use –soil erosion– is linked to grid electricity. In contrast, the burdens on terrestrial eutrophication and acidification are related to other activities, followed by direct NOx emissions and grid electricity use. Similarly to SDG 14, burdens increase in the carbon-neutral solutions due to the high power requirements (further details on the links with the bespoke mix in **Figure S1**). Finally, terrestrial eutrophication improves due to lower NO_x direct emissions, as in photochemical ozone formation and marine eutrophication.

2.4. Sensitivity analysis

In this section, we present the sensitivity analysis results, which identify the most influential parameters of the carbon-neutral chemical system and quantify their effect on the economic and environmental performance.

Uncertain parameters and variation rate

In the main manuscript, we showed that the $CHEMZERO$ design is more expensive than the BAU (by 16.5 and 33.0%), while its $SUST_{index}$ is significantly lower (from 9.2 to 7.3 and 5.9). In this section, we explored how the latter values change under uncertainty. To this end, we vary key uncertain parameters, either one by one or altogether, recalculating the performance of the optimal designs in each case (cost and $SUST_{index}$). More precisely, we perform two analyses, one by changing parameters of similar nature (grouped into the same uncertainty block) or simultaneously, and a second analysis varying only individual parameters at a time (i.e., the capacity factor and LCOE of an individual technology). The first analysis results are displayed using violin plots, while for the second we use polylines figures. This analyses are performed for the two carbon-neutral solutions reported in the main manuscript. Namely, the uncertain parameters blocks are the following:

1. The chemicals' production costs of 27 out of the 29 processes listed in **Table 1** (we investigate the electrolysis and DAC process separately). We exclude the costs related to non-primary feedstock (i.e., H_2 and CO_2) and their energy supply. These expenditures cover capital and operational costs uncertainties, which are region-dependent.
2. The power system backup reserves parameter, BACKUP.
3. The capacity factor of the power technologies listed in **Table 2**.
4. The levelised cost of electricity (LCOE) of the power technologies listed in **Table 2**.
5. The production cost of eH_2, excluding the contributions from the consumption of electricity (i.e., electrolyser costs).
6. The efficiency of the electrolysis step.
7. The DAC production cost, excluding the cost related to heat and electricity consumption, which are analysed separately (8 and 9).
8. The DAC electricity consumption.
9. The DAC heat consumption.
10. Simultaneous variation of the latter nine sets of parameters.

We vary the latter parameters by applying a Monte Carlo sampling on a set of uniform distributions modelling them (we chose the uniform distributions due to lack of detailed uncertainty data on all the parameters, although other distributions could have been defined instead). The uniform distributions are built considering lower and upper bounds equal to ±20% of the nominal values, except for the electrolyser efficiency, for which we use ±10% bounds since the base case is already optimistic (80% of the LHV, see **Section 1.4**). The value of the capacity factor cannot exceed 1.0, so we cap the upper capacity factor accordingly. We generate 1000 scenarios per parameter and technology for each set of parameters to ensure a high confidence level (99.9%). Subsequently, we run and solve the linear programming model for each of them to recalculate the total cost and the sustainability index.

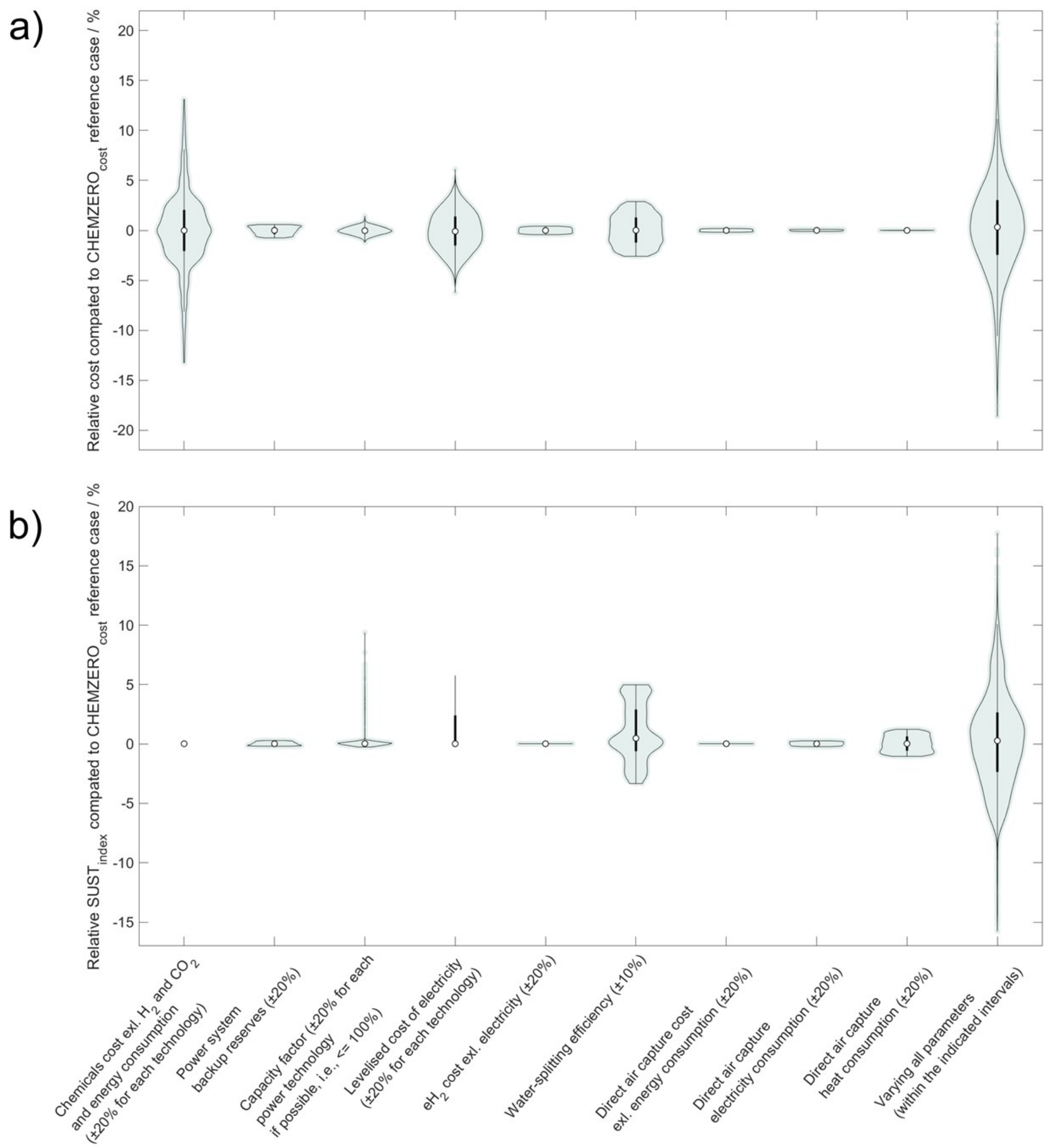

Figure S3. Sensitivity analysis of the $CHEMZERO_{cost}$ design. Each violin depicts the probability distribution of the (a) optimal cost and (b) the accompanied $SUST_{index}$ (expressed as a percentage) when uncertainty is considered. The violin's width reflects the solutions' frequency (i.e., number of scenarios).

Sensitivity $CHEMZERO_{cost}$

The analysis revealed that the most critical parameters are the chemicals production cost, the LCOE of the power technologies, and the water-splitting efficiency, leading to variations in the cost of ±13.0, 5.2, and 2.8%, respectively (**Figure S3a**). Nonetheless, the width of the violin plot indicates that cost variations beyond ±5% are much less likely for the first uncertain parameter. In contrast, the width of the violin plots when investigating the LCOE and the capacity factors is fairly constant within the confidence interval.

Moving to the $SUST_{index}$ in the same solution (**Figure S3b**), we find that only the water-splitting efficiency and, to a lesser extent, the DAC heat consumption influence the transgression levels

significantly. The $SUST_{index}$ value decreases by 3.3% when the electrolyser efficiency increases (+10%, upper bound of the range), while there is a higher impact for a lower efficiency (−10%, lower bound of the range) equal to +5.0%. The latter behaviour emerges due to active constraints and parameters associated with the power system, such as backup reserves, source availability, and growth rate.

Regarding the effect of varying all uncertain parameters simultaneously, we find that even though the total cost might lie within the range of ±20.0%, most scenarios fall within a shorter interval (±5.0%). This might be due to the fact that uncorrelated distributions are considered, so variations in one parameter might be offset by changes in others. At the same time, the response of the system's $SUST_{index}$ is smaller, equal to around ±10.0% of the reference scenario.

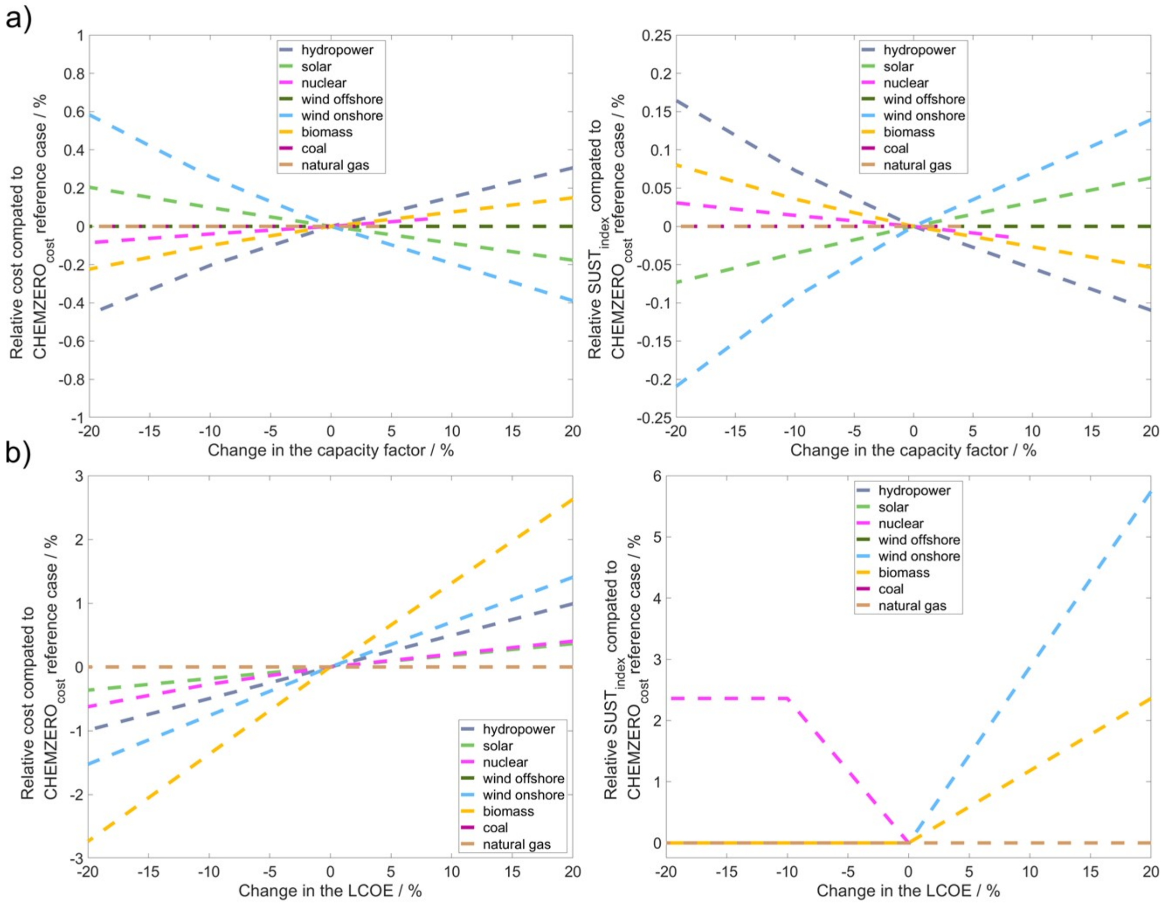

Figure S4. Sensitivity analysis of the cost (left) and the accompanied $SUST_{index}$ (right) of the $CHEMZERO_{cost}$ design (expressed as a percentage) when varying (a) the capacity factor and (b) LCOE of the power technologies individually.

Secondly, we analyse the influence of uncertainties in the individual power technologies on the $CHEMZEO_{cost}$ design to highlight their influence within their distinct uncertain block (**Figures S4-S12**). To this end, we report the solutions corresponding to 5 scenarios with an equidistant discretisation of the range previously described.

We observe that the capacity factor of wind power and hydropower reservoirs are the most influential technologies of this uncertain block (**Figure S4a**). Furthermore, the capacity factor shows a non-linear influence on the cost and $SUST_{index}$, and a converse trend between

dispatchable and non-dispatchable power technologies, which are both related to the constraint of backup reserves. Notably, burden shifting in specific LCA indicators still occurs, as discussed in the main manuscript, when varying the capacity factor (see **Figure S5**).

The influence of the LCOE is higher (**Figure S4b**), where the cost of the biomass power plants is the most influential parameter on the system's total cost (~±3.0%), followed by wind onshore (~±1.5%), and hydropower reservoirs (~±1.0%). The $SUST_{index}$ value increases with lower LCOE for nuclear power, while it remains unchanged when the remaining power technologies exhibit lower costs. In addition, with higher LCOE for the power technologies, we observe that the wind onshore and bioenergy are the most influential parameters on the $SUST_{index}$. Regarding the insight of burden shifting occurrence, as before, it remains valid when varying the LCOE (see **Figure S6**).

Note that the system adjusts the selection of technologies when we vary the uncertain parameters, i.e., select different power or chemical technologies (such as the CCU to fossil-based petrochemical ratio). These specific trends are not discussed in detail, since the solutions found follow the same general patterns (**Figure S7**).

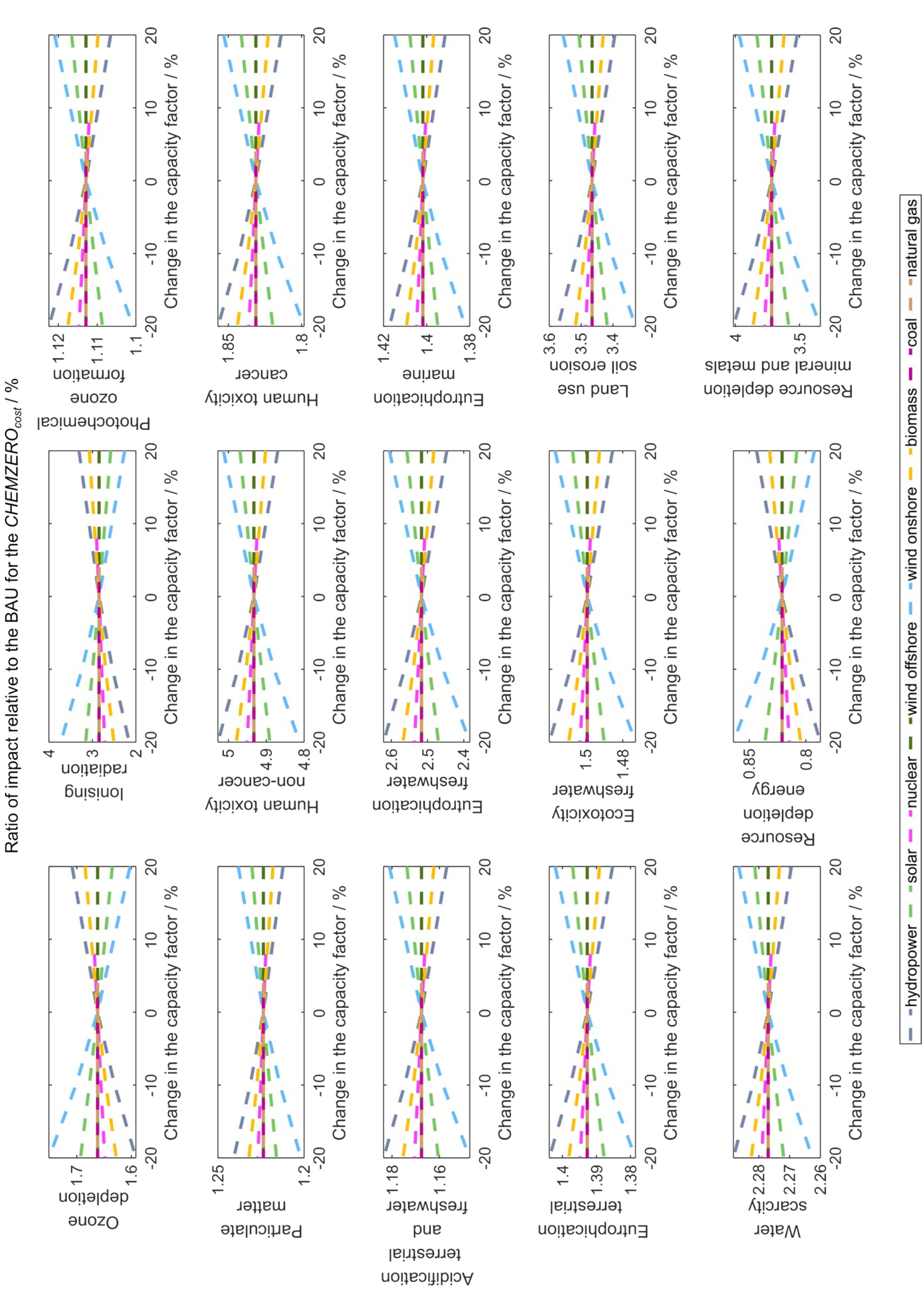

Figure S5. Sensitivity analysis of the individual impacts of the $CHEMZERO_{cost}$ design (expressed as a ratio relative to the BAU performance) when varying the capacity factor of the power technologies individually. A value greater than 1 means that the performance of the system worsens relative to the BAU.

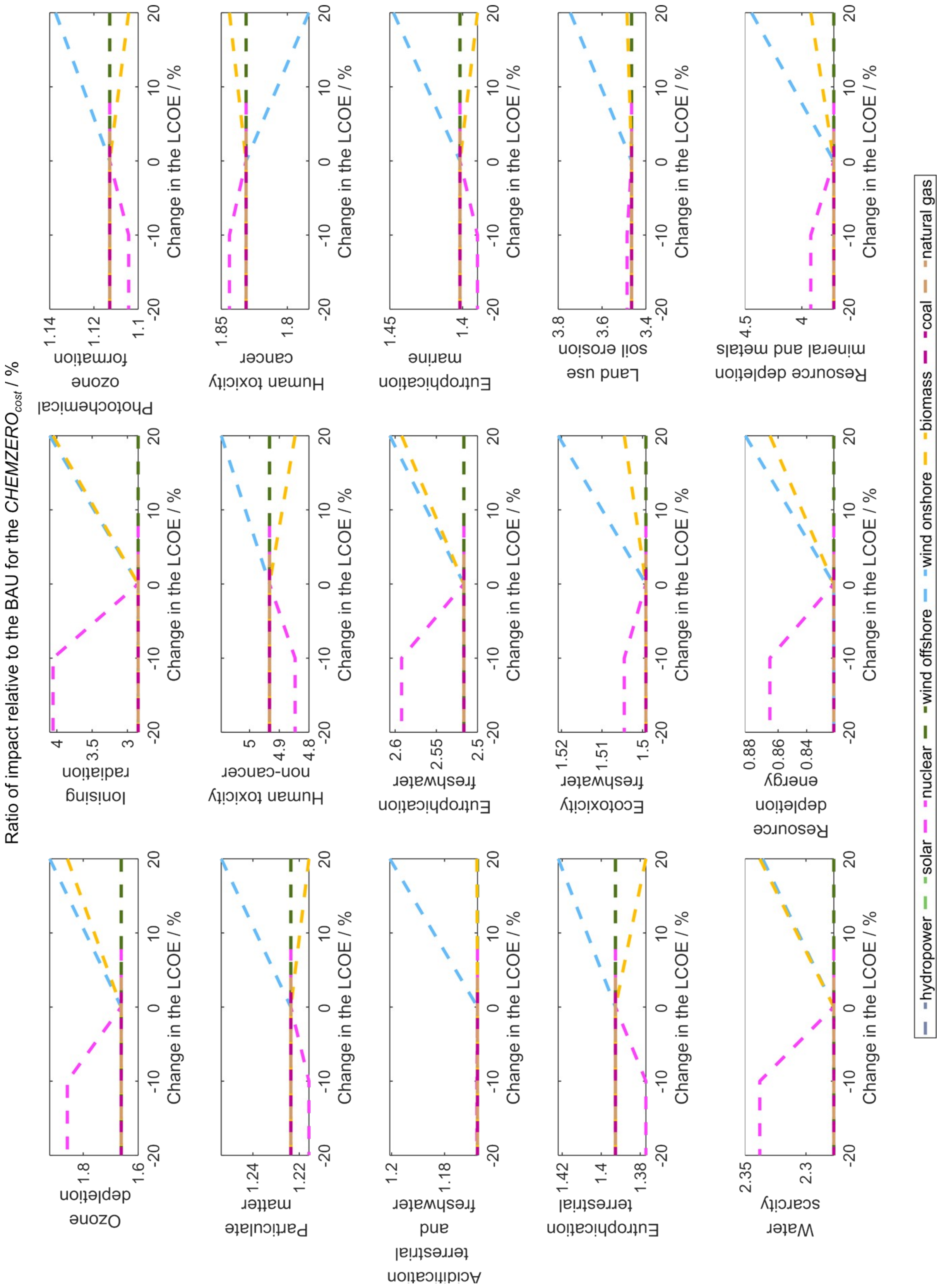

Figure S6. Sensitivity analysis of the individual impacts of the $CHEMZERO_{cost}$ design (expressed as a ratio relative to the BAU performance) when varying the LCOE of the power technologies individually. A value greater than 1 means that the performance of the system worsens relative to the BAU.

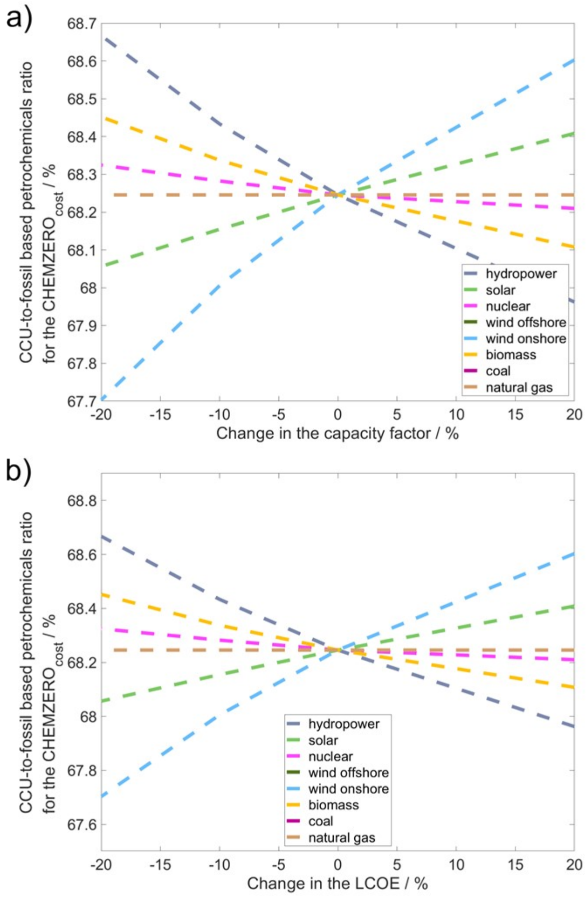

Figure S7. Sensitivity analysis of the ratio between the CCU and fossil-based petrochemicals of the $CHEMZERO_{cost}$ design (expressed in %) when varying (a) the capacity factor and (b) the LCOE of the power technologies individually.

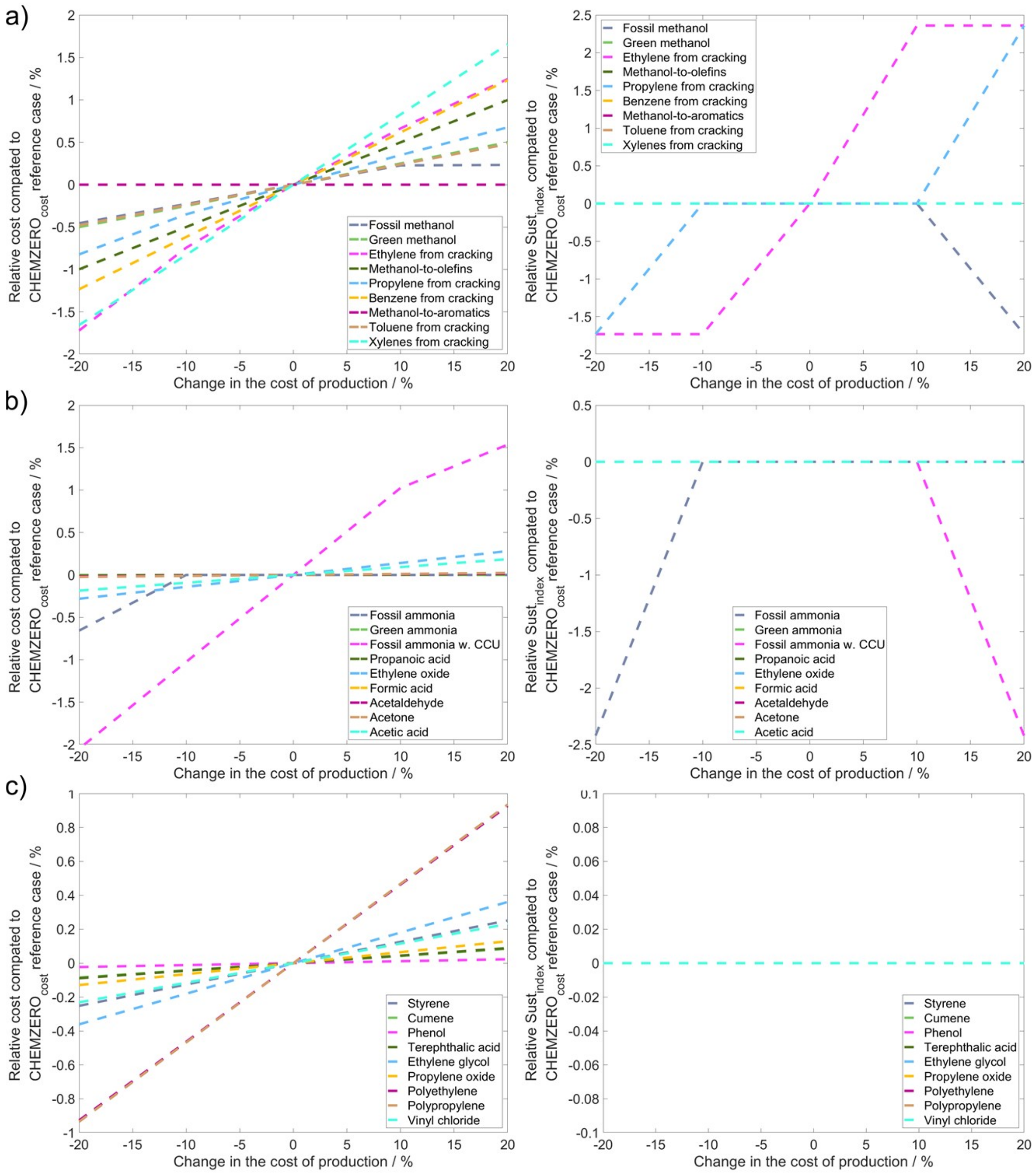

Figure S8. Sensitivity analysis of the cost (left) and the accompanied $SUST_{index}$ (right) of the $CHEMZERO_{cost}$ design (expressed as a percentage) when varying the production costs of the chemical production technologies individually. We split the technologies into three plots (**a-c**) for readability purposes.

We discuss next the impact of varying individually the chemicals' production costs. Firstly, we observe that petrochemicals and polymers (**Figure S8a**) influence the cost the most due to their high production volume. Furthermore, we observe that lower costs of fossil-based ammonia (**Figure S8b**) have a negligible effect on the total cost, yet they lower the value of $SUST_{index}$. Notably, the latter $SUST_{index}$ decrease is due to the lower CCU deployment as shown in **Figure 9**, owing to the substitution of the CO_2 source from ammonia to DAC plants. Finally,

for those technologies that cannot be substituted (**Figure S8c** left, e.g., chemicals vinyl chloride), we observe no influence on the $SUST_{index}$ and individual LCA metrics (**Figure 12**).

All in all, the influence of the production cost on the LCA metrics is depicted in **Figures 10-12**, where we obtain similar insights concerning burden shifting as in the reference scenario.

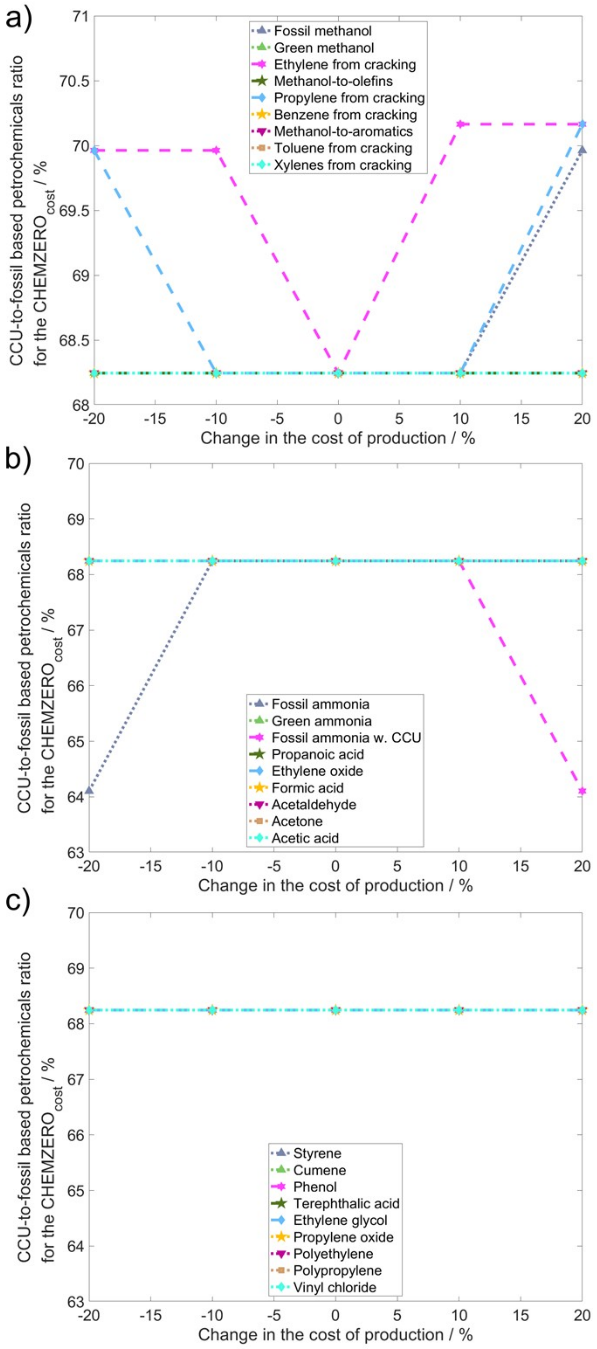

Figure S9. Sensitivity analysis of the CCU to fossil-based petrochemicals ratio of the $CHEMZERO_{cost}$ design (expressed as a %) when varying the production expenditures of the chemical production technologies individually. We split the technologies into three plots (**a-c**) for readability purposes.

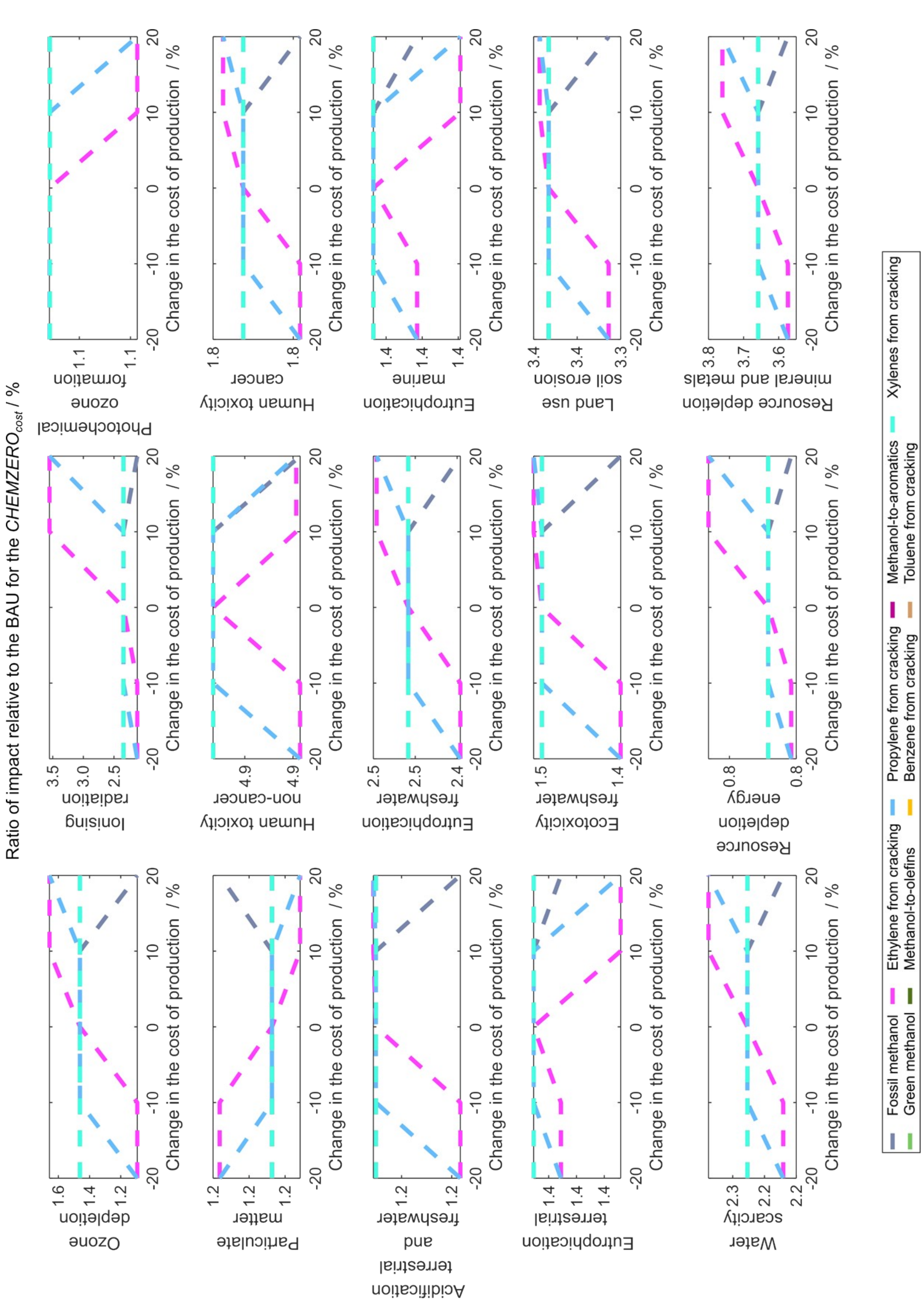

Figure S10. Sensitivity analysis of the individual impacts of the $CHEMZERO_{cost}$ design (expressed as a ratio relative to the BAU performance) when varying the production expenditures of the chemical production technologies individually. We split the technologies into three plots (**Figure S10-S12**) for readability purposes.

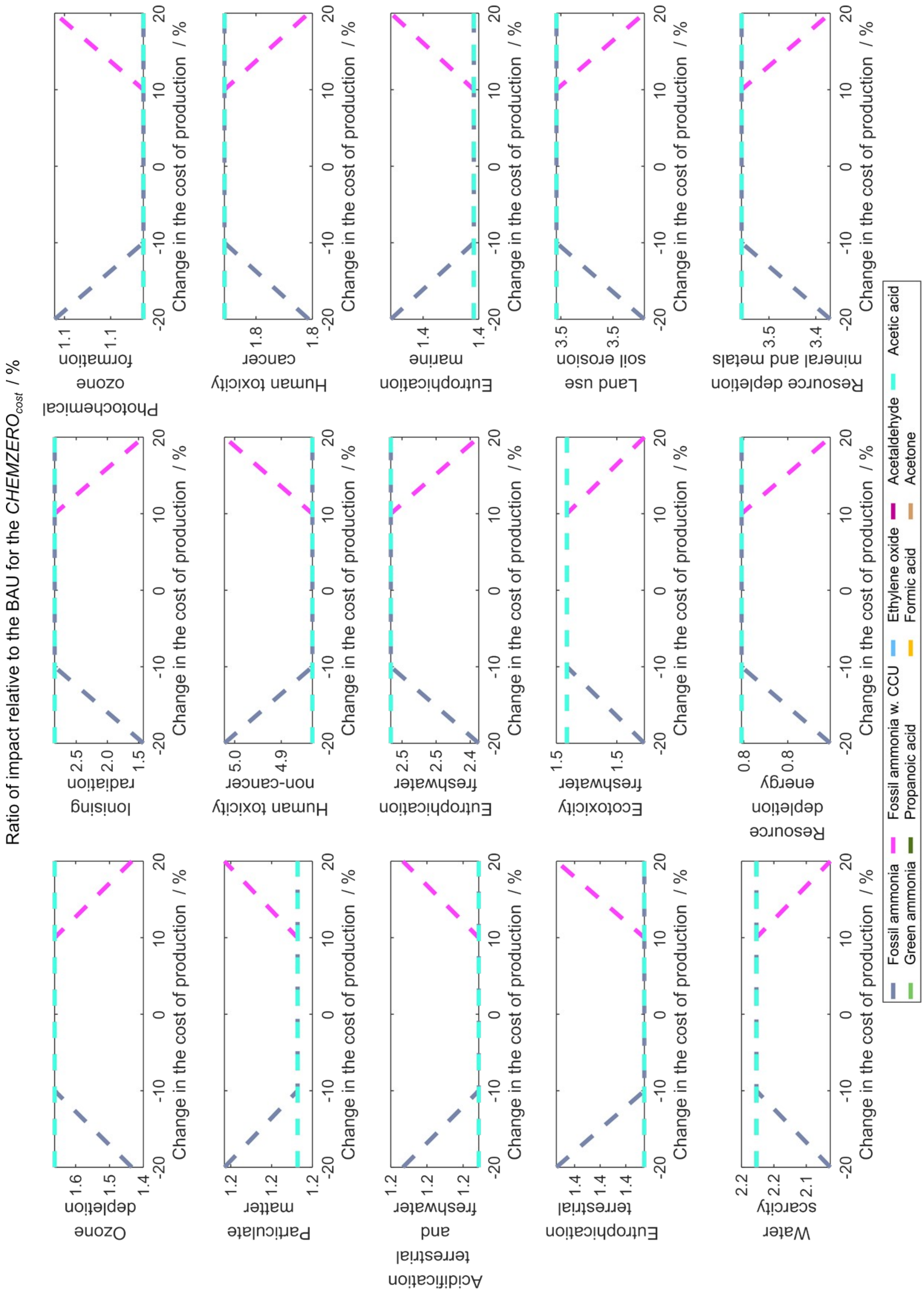

Figure S11. Sensitivity analysis of the individual impacts of the $CHEMZERO_{cost}$ design (expressed as a ratio relative to the BAU performance) when varying the production expenditures of the chemical production technologies individually. We split the technologies into three plots (**Figure S10-S12**) for readability purposes.

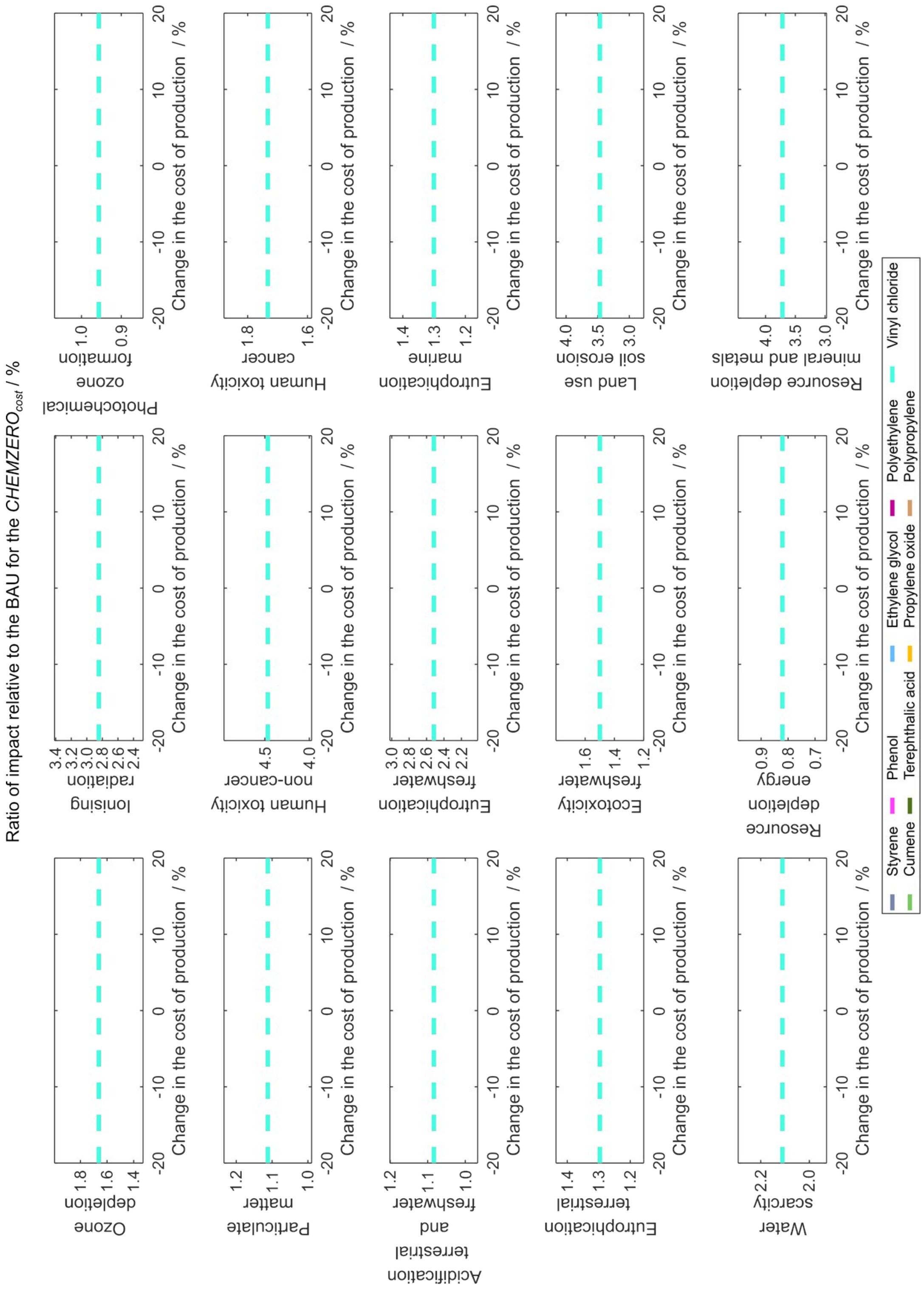

Figure S12. Sensitivity analysis of the individual impacts of the $CHEMZERO_{cost}$ design (expressed as a ratio relative to the BAU performance) when varying the production expenditures of the chemical production technologies individually. We split the technologies into three plots (**Figure S10-S12**) for readability purposes.

Sensitivity $CHEMZERO_{sust}$

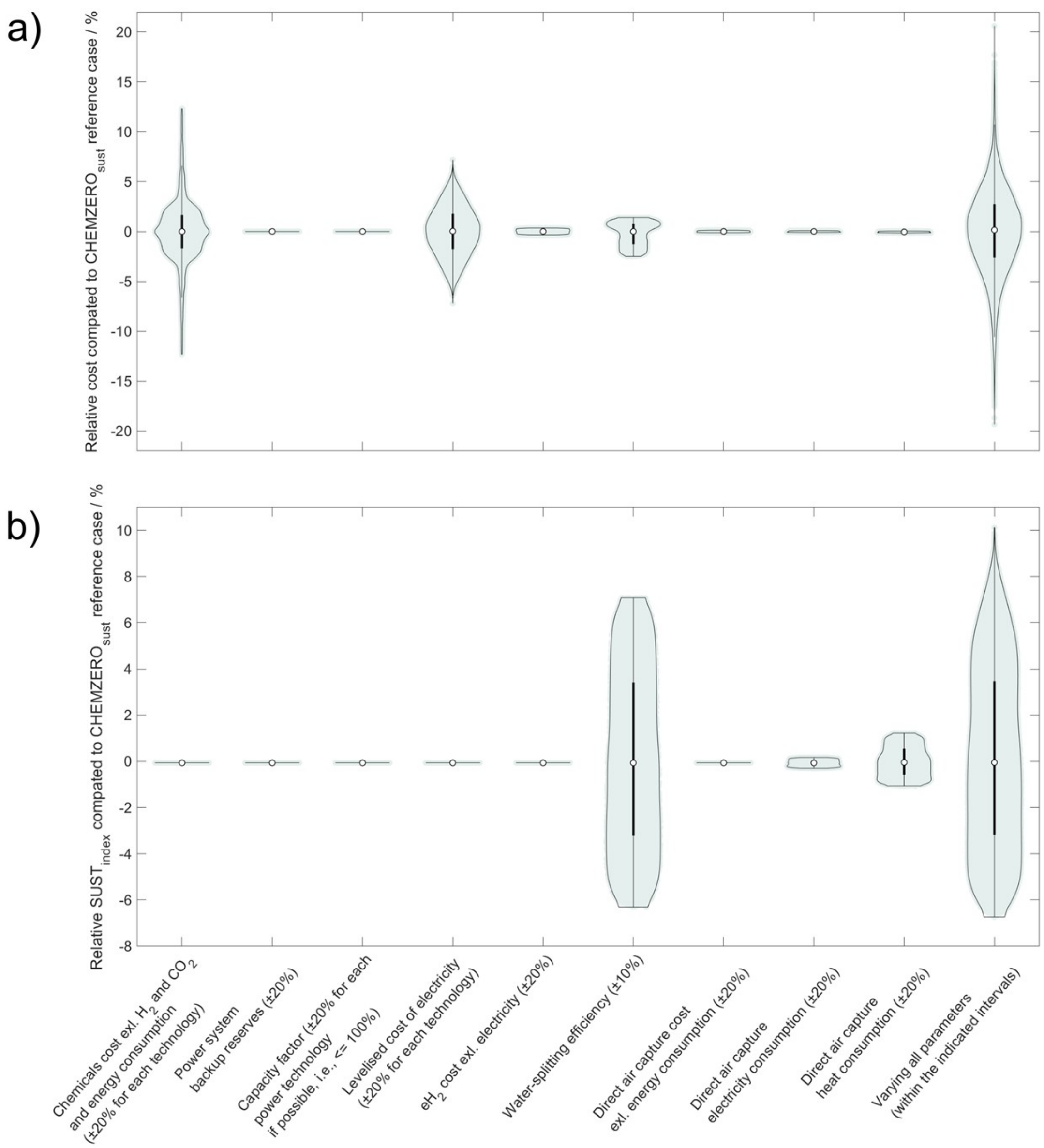

Figure S13. Sensitivity analysis of the cost and environmental performance of the $CHEMZERO_{sust}$ design when varying uncertain parameters within a given interval. Each violin depicts the probability distribution of (a) the accompanying total cost for (b) the optimal $SUST_{index}$ (expressed as a percentage) when uncertainty is considered. The violin's width reflects the solutions' frequency (i.e. number of scenarios).

The analysis revealed that in terms of the total cost the influence of the LCOE, chemicals' production cost and the electrolyser efficiency follows a similar trend to that observed in the sensitivity of $CHEMZERO_{cost}$ (**Figure S13a**). In contrast to the minimum cost analysis, 6 out of the 9 uncertain blocks affect the system's cost only marginally. Regarding the BACKUP parameter, and the capacity factor and LCOE of power technologies, this behaviour arises mainly due to the allocation of different technologies in the backup reserves. At the same time, the effect of

the DAC's utilities is negligible since the capacity of CCU technologies is significantly lower in the $CHEMZERO_{sust}$ compared to $CHEMZERO_{cost}$.

Moving to the $SUST_{index}$ in the same solution, we find that it is influenced significantly when varying the electrolyser efficiency (ranging between -6.3 to 7.1% compared to the reference), while the heat and electricity consumption of the DAC process follows, with a negligible influence (~±1.0 and ~±0.2%, respectively). Regarding the remaining uncertainty blocks, we observe that the optimal $SUST_{index}$ is not influenced by variations of the 6 (out of 9) uncertain parameters (**Figure S13b**), since they are mainly related to economics rather than environmental performance. Besides, recall that we first optimise the transgression level and later post-process the solution to find the minimum cost for the optimal $SUST_{index}$ value.

Overall, when all uncertain parameters are varied simultaneously, the $SUST_{index}$ value ranges between -6.7 to +10% relative to the reference design. As before, we find that the total cost might lie within the range ±20.0%, with a higher frequency within a shorter interval (±5.0%).

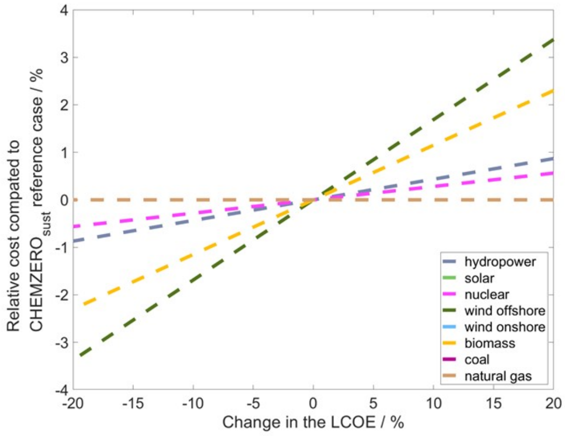

Figure S14. Sensitivity analysis of the cost of the $CHEMZERO_{sust}$ design (expressed as a percentage) when varying the LCOE of power technologies individually.

Regarding the individual variation of the LCOE in $CHEMZERO_{sust}$ design (**Figure S14**), we observe a similar trend as in $CHEMZERO_{cost}$. Nonetheless, the wind offshore emerges as one of the most critical technology, followed by bioenergy, hydropower reservoir, and nuclear. Finally, the sensitivity depicted in **Figure S15**, unlike **Figure S5**, shows a linear system response concerning production cost variations since the total cost is the secondary objective of $CHEMZERO$.

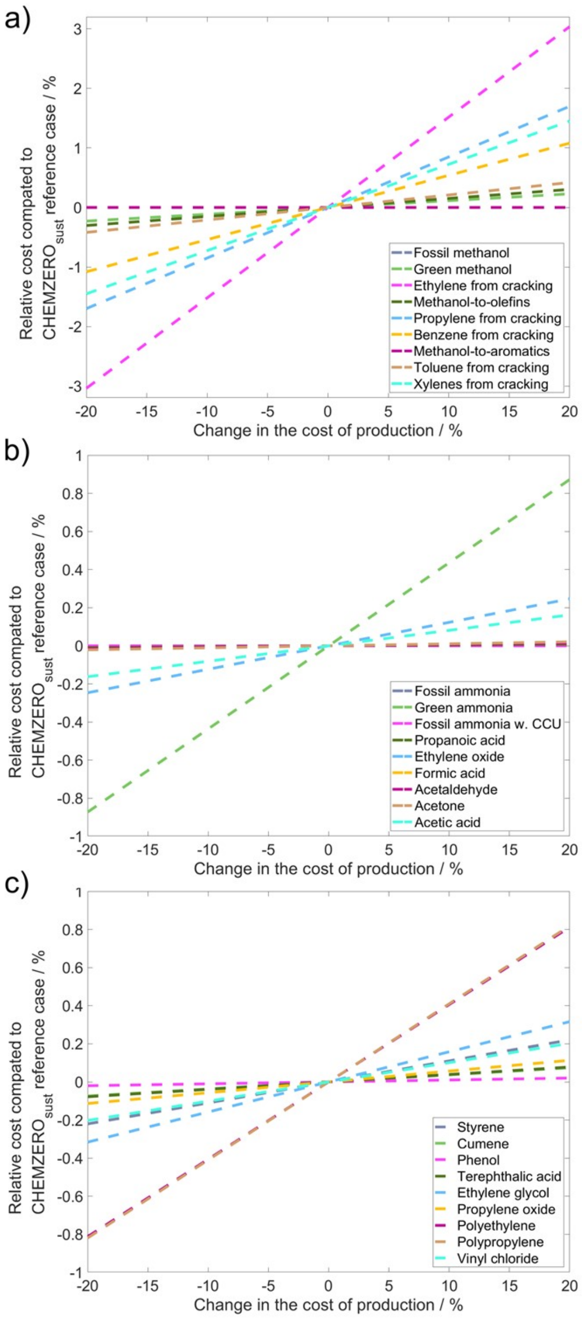

Figure S15. Sensitivity analysis of the cost of the $CHEMZERO_{sust}$ design (expressed as a percentage) when varying the production expenditures of the chemical production technologies individually. We split the technologies into three plots (**a-c**) for readability purposes.

2.5. Price elasticity of demand

Chemicals in the $CHEMZERO$ solution are more expensive than in the BAU, which could reduce their demand based on the price elasticity of chemicals (**Table 8**), defined as follows:

$$\varepsilon = \begin{cases} \dfrac{\partial Quantity / Quantity}{\partial Price / Price} & ,if\ continuous \\ \dfrac{\dfrac{Quantity_2 - Quantity_1}{Quantity_1}}{\dfrac{Price_2 - Price_1}{Price_1}} = \dfrac{\%\ change\ in\ Quantity}{\%\ change\ in\ Price} & ,if\ discrete \end{cases} \tag{21}$$

Essential goods, i.e., bread, electricity, and gasoline, tend to be less elastic. In contrast, luxury goods tend to be more sensitive to price changes, i.e., electronics and restaurant meals.

Table 8 Values, classification, and meaning for the price elasticity of demand value[42]

Value	Classification	Meaning
ε = 0	Perfectly inelastic	The quantity demanded is entirely insensitive to the price.
0 < ε < -1	Inelastic	Quantity demanded is relatively insensitive to price.
ε = -1	Unitary elastic	A percentage increase in quantity demanded equals a percentage decrease in price.
-1 < ε < −∞	Elastic	Quantity demanded is relatively sensitive to price.

Hence, more expensive routes are expected to lead to lower demands. While subsidies or a carbon tax could increase the economic appeal of a CO_2-based chemical sector, the assumption of a constant final demand still needs to be evaluated. Therefore, in this section, we discuss the price-demand relation in the investigated $CHEMZERO$ designs.

Discussion

The price elasticity of consumer demand products can be quantified from real-world observations[43,44] or through computable general equilibrium (CGE) models.[45] One might assume that the chemical sector is relatively inelastic since it is a key driver of productivity and economic growth. Besides, chemicals are transformed into a myriad of essential products in everyday life. For example, a study estimated that nitrogen and phosphorus, in contrast to potash fertilisers, are inelastic.[46] Furthermore, chemical production is also driven by the consumption of inelastic commodities, such as natural gas and crude oil.[43,44] Notably, a CGE study based on Finnish households estimated that the price elasticity for chemicals ranges between -0.45 to -0.25,[45] so they could be considered inelastic.

To assess the inelastic behaviour assumption, we carry out a post optimal quantification of the demand response in the next section based on our calculations in each solution. We stress that, according to the ISO standards, the assumption of constant final demand is vital to

compare the alternative designs fairly, so price demand curves should not be modelled explicitly in CHEMZERO as they would lead to different demands depending on the costs.

Regression of the price elasticity of demand

To perform our analysis, we first regress a demand function with real-world data to model the relationship between prices and demand of chemicals. Specifically, we follow here the work by J.C. B. Cooper[44]:

$$D_{t,i} = \alpha_i \cdot Price_{t,i}^{\beta_i} \cdot GDP_t^{\gamma_i} \cdot D_{t-1,i}^{\delta_i},\ \forall t,i \tag{22}$$

where $D_{t,i}$ (expressed in Mt) and $Price_{t,i}$ (expressed in \$ t[-1]) are the demand and price of chemical i at year t, respectively, while GDP_t is the per capita real world gross domestic product (GDP) at year t, expressed in \$ per person. Finally, $\alpha, \beta, \gamma,$ and δ are coefficients to be estimated based on the following real-world data spanning several years: (i) demand,[47] (ii) prices,[48] (iii) world GDP per capita,[49] and (iv) inflation data.[50]

Due to data gaps, we regress **Eq. 22** by using methanol as a reference chemicals capturing the elasticity behaviour of the other chemicals. The latter parameters were regressed for the period $2011 \le t \le 2021$, leading to the following values:

$$D_{t,i=MeOH} = 9.17 \cdot 10^{-4} Price_{t,i=MeOH}^{-0.14} GDP_t^{0.92} D_{t-1,i=MeOH}^{0.85},\ \forall t \in [2011,2021] \tag{23}$$

Notably, according to the original work, methanol could be considered relatively inelastic since the estimated short-run elasticity of demand is equal to $\beta = -0.14$, and the long-run elasticity is $\beta/(1-\delta) = -0.92$.[44] The latter values indicate a slow demand response in the short term, and a more elastic response in the long term. Moreover, the short term elasticity close to zero highlights the difficulty of substituting the consumption within a short interval, while the long term one indicates that shifting to other alternatives within an adequate time horizon is more likely.

Validation based on the expected demand for methanol

We then re-calculate the demand for methanol in 2050 using **Eq. 23** and the expected BAU price and GDP for 2050, and compare it with the original value we considered in the calculations (which was taken from an IEA[22] report). **Eq. 23** requires the demand for 2049, which we can estimate as follows:

$$D_{t-1,i} = D_{t_{ref},i} \prod_{t'=t_{ref}}^{t-1} \left[1 + G_{t',i}\right],\ \forall t \in [2011,2050], i \tag{3}$$

where $D_{t_{ref},i}$ is the demand for chemical i at the reference year $t_{ref} = 2010$, and $G_{t,i}$ is the annual growth rate of the demand for product i at year t.

Based on the latter, the calculated methanol demand based on its price elasticity is 196.6 Mt, which is almost the same as the original value assumed in this study (196.9 Mt). This means that the elasticity model is rather accurate.

Recalibration for the other chemicals

The validated elasticity model might not be fully accurate for the remaining chemicals. Taking methanol as reference chemical, we assume that the elasticity parameters (β and γ) and the

productivity parameter (δ) are the same across chemicals, but re-calibrate the model to adjust parameter a_i for each of them. This is done using their expected final demand for 2050 and the BAU prices, obtaining a refined elasticity model for each of them. This model will be finally used to predict their demand from their production costs.

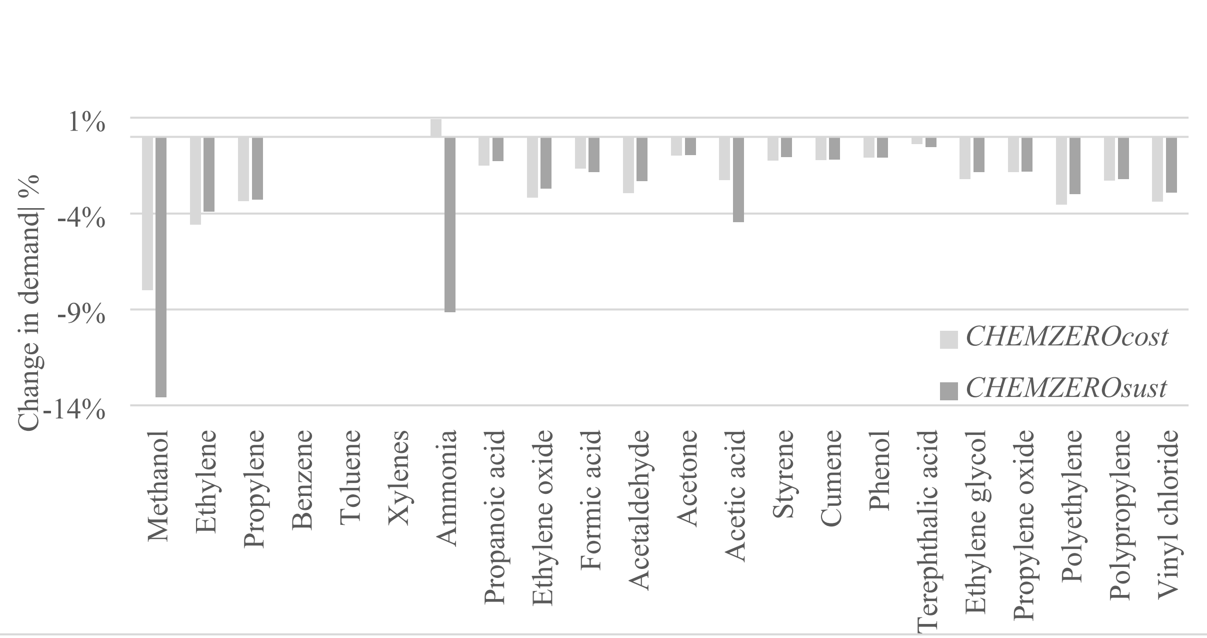

Figure S16. Percentage change in demand for the $CHEMZERO_{cost}$ and $CHEMZERO_{sust}$ designs, recalculated considering prices elasticities.

Post-optimal analysis

Figure S16 depicts each product's percentage change in demand within the chemical system relative to the original value based on an inelastic behaviour. In $CHEMZERO_{cost}$, we observe that ammonia production generates value by selling CO_2 within the network for CCU. Therefore, the decrease in ammonia's price (shifting from plants that vent the CO_2) will lead to a marginal increase in the expected demand (**Figure S16**). In contrast, the sharp transition to a Haber-Bosch process based on eH_2 in $CHEMZERO_{sust}$ shows a converse trend due to the significant price increase. Regarding the organic chemicals, we observe that methanol experiences the most significant change in demand (8.0 – 13.6% demand reduction). The remaining products' demand changes much less, within the range of 0.4 – 4.6%, with terephthalic acid and ethylene at the lower and higher ends, respectively.

Overall, the assumption of constant final demand results in the overestimation of cost and impacts, yet demand changes are <14% in all the chemicals, and <5% in many of them. Finally, as mentioned earlier, subsidies could mitigate to some extent this change in demand.

2.6. Expanding the power technologies portfolio

In this section, we expand the portfolio of power technologies to include concentrated solar power, solar photovoltaics, and wind onshore integrated with a utility-scale battery (as described in **Section 1.3**).

Discussion

When adding the new technologies, the obtained chemical system designs are similar to the ones of the reference counterpart (**Figure S17**). Specifically, the cost of $CHEMZERO_{cost}^{expanded}$ decreases by 0.2% and its $SUST_{index}$ increases by 12.9% (8.24 vs. 7.30). This is due to the fact that the model decides to (i) avoid nuclear power, (ii) generate power with solar photovoltaics integrated with utility-scale batteries, and (iii) doubling solar photovoltaic power without utility-scale batteries compared to the reference case (**Figure S18**). The latter changes lead to a power system with higher carbon footprint when compared to the reference case (−0.05 vs. −0.11 $kgCO_{2eq}$ kWh^{-1}). Finally, in the sustainable solution, the cost increases slightly (+0.2%) while leading to a 2.3% decrease in the sustainability index (5.76 vs. 5.90). Nuclear power is almost halved in $CHEMZERO_{sust}^{expanded}$ and it is substituted with concentrated solar power, while the carbon footprint of the power system is almost identical to that in the reference case (**Figure S18)**.

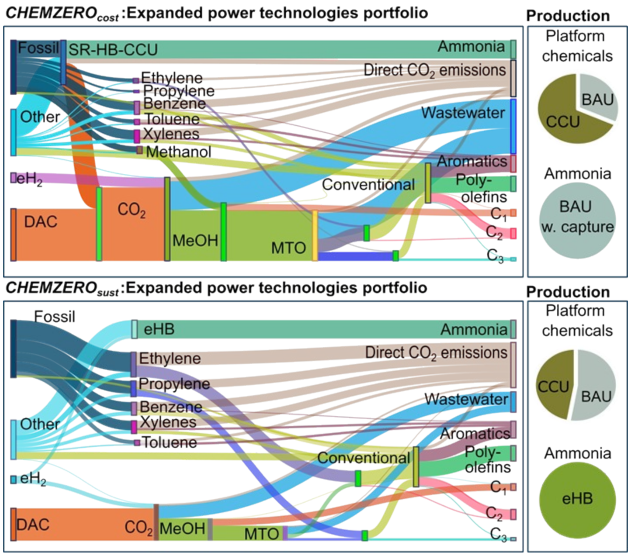

Figure S17. Total mass flows, gate-to-gate, within the chemical system of the two expanded optimal solutions. The objective function influences the technologies' selection drastically. Both solutions reduce the mass of fossil-based feedstock (e.g., oil, coal, natural gas, and shale gas) and the direct CO_2 emissions compared to the BAU. Furthermore, the optimal solutions offset the fossil-based emissions by utilising carbon-negative electricity (embodied in the eH_2) and CO_2 from air.

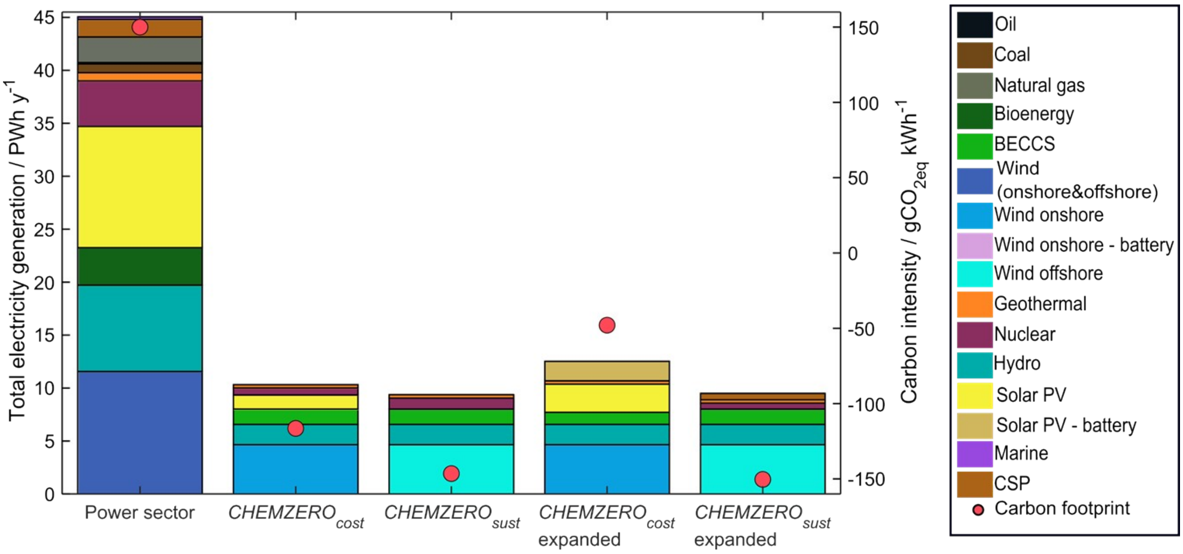

Figure S18. Annual power generation and breakdown for the global anthropogenic activities in 2050, as forecasted in the world energy outlook, and power generation from the bespoke mixes of the $CHEMZERO_{cost}$ and $CHEMZERO_{sust}$ for the reference and expanded portfolio (primary axis) their respective carbon footprint (secondary axis).

Overall environmental performance

Figure S19 provides the transgression level and breakdown for the reference case and expanded portfolio. On the one hand, the $CHEMZERO_{cost}^{expanded}$ solution performs worse in 13 out of the 16 LCA indicators when compared to $CHEMZERO_{cost}$, and better in energy resources depletion and ionising radiation. On the other hand, $CHEMZERO_{sust}^{expanded}$ and $CHEMZERO_{sust}$ perform almost in the same way, except once again in energy resources depletion and ionising radiation impacts, whose transgression level diminishes by 6.9 and 33.6%, respectively.

Life on land goal (SDG 3)

The cost-effective carbon-neutral design of the $CHEMZERO_{cost}^{expanded}$ portfolio worsens all metrics related to SDG 3, with the non-cancer and cancer human toxicity impact being higher by 15.2 and 13.8%, respectively (relative to $CHEMZERO_{cost}$). Furthermore, the particulate matter and photochemical ozone formation impact also increase (by 10.3 and 5.9%, respectively). Finally, $CHEMZERO_{cost}^{expanded}$ leads to a lower ionising radiation impact (by -49.4%) compared to its reference counterpart. The latter behaviour occurs mainly due to the higher consumption of power within the system (12.5 vs. 10.3 PWh, see **Figure S18**). The $CHEMZERO_{sust}^{expanded}$ design shows a reduction in ionising radiation impacts (by 33.6%), while worsening only slightly the other metrics linked to SDG 3. The non-cancer and cancer human toxicity is higher by 0.8 and 2.1%, while higher particulate matter and photochemical ozone formation impacts are also obtained (by 1.0 and 0.7%, respectively).

Life on land goal (SDG 6)

The $CHEMZERO_{cost}^{expanded}$ design performs worse than the reference base case in this SDG, leading to 10.5 and 14.4% higher water scarcity and freshwater ecotoxicity impact. Once more, we observe that $CHEMZERO_{sust}^{expanded}$ shows an almost identical SDG 6 performance when compared to $CHEMZERO_{sust}$.

Climate action goal (SDG 13)

The expanded designs perform better in the energy resources depletion metric of the SDG 13 metrics while attaining the carbon neutrality target (by 11.7 and 6.3% for the minimum cost and sustainability index design, respectively). Moreover, $CHEMZERO_{cost}^{expanded}$ shows an almost 2.5-fold increase in ozone depletion, while $CHEMZERO_{sust}^{expanded}$ reduces the impact of this metric by 11.7%.

Life on land goal (SDG 14)

The $CHEMZERO_{cost}^{expanded}$ design performs worse than the reference base case in this SDG. Besides, the impact on freshwater and marine eutrophication is higher by 55.5 and 8.4%, respectively. In contrast, $CHEMZERO_{sust}^{expanded}$ provided a design which lowers both metrics when compared to its reference counterpart (lower impact by 55.4 and 16.9%, following the same sequence as before)

Life below water goal (SDG 15)

The depletion of minerals and metals suffers significant burden-shifting in the $CHEMZERO_{cost}^{expanded}$ relative to its reference counterpart (an almost 2-fold increase), and occupies 20.3% of the SOS. Even though the remaining categories of this SDG show higher impacts than the reference counterpart, their transgression levels are low (≤2.3% of the SOS). Notably, we observe that $CHEMZERO_{sust}^{expanded}$ shows the same SDG 15 performance as $CHEMZERO_{sust}$.

Chemical system design

In $CHEMZERO_{cost}^{expanded}$ the model leads to higher consumption of eH_2 and CO_2 from DAC relative to the reference case, equal to 280.7 vs. 231.7 MtH_2 and 1.5 vs. 1.2 $GtCO_{2eq}$, respectively. The additional amount of the latter precursors leads to a higher substitution of olefins produced from the MTO process compared to $CHEMZERO_{cost}$ (87.7 vs. 75.0%). Moreover, the higher consumption of CO_2 from the air in the expanded portfolio leads to a negative contribution of the net CO_2 direct emissions, as depicted in the impacts breakdown of **Figure S17** (utilised CO_2 from air > CO_2 directly emitted). Notably, a higher CO_2 feedstock from the air is necessary because the power system's carbon footprint is significantly higher than that of the reference power mix (−0.05 vs. −0.11 vs. $kgCO_{2eq}$ kWh^{-1}). Finally, the mass flow changes in the $CHEMZERO_{sust}^{expanded}$ are almost the same as those in the reference counterpart.

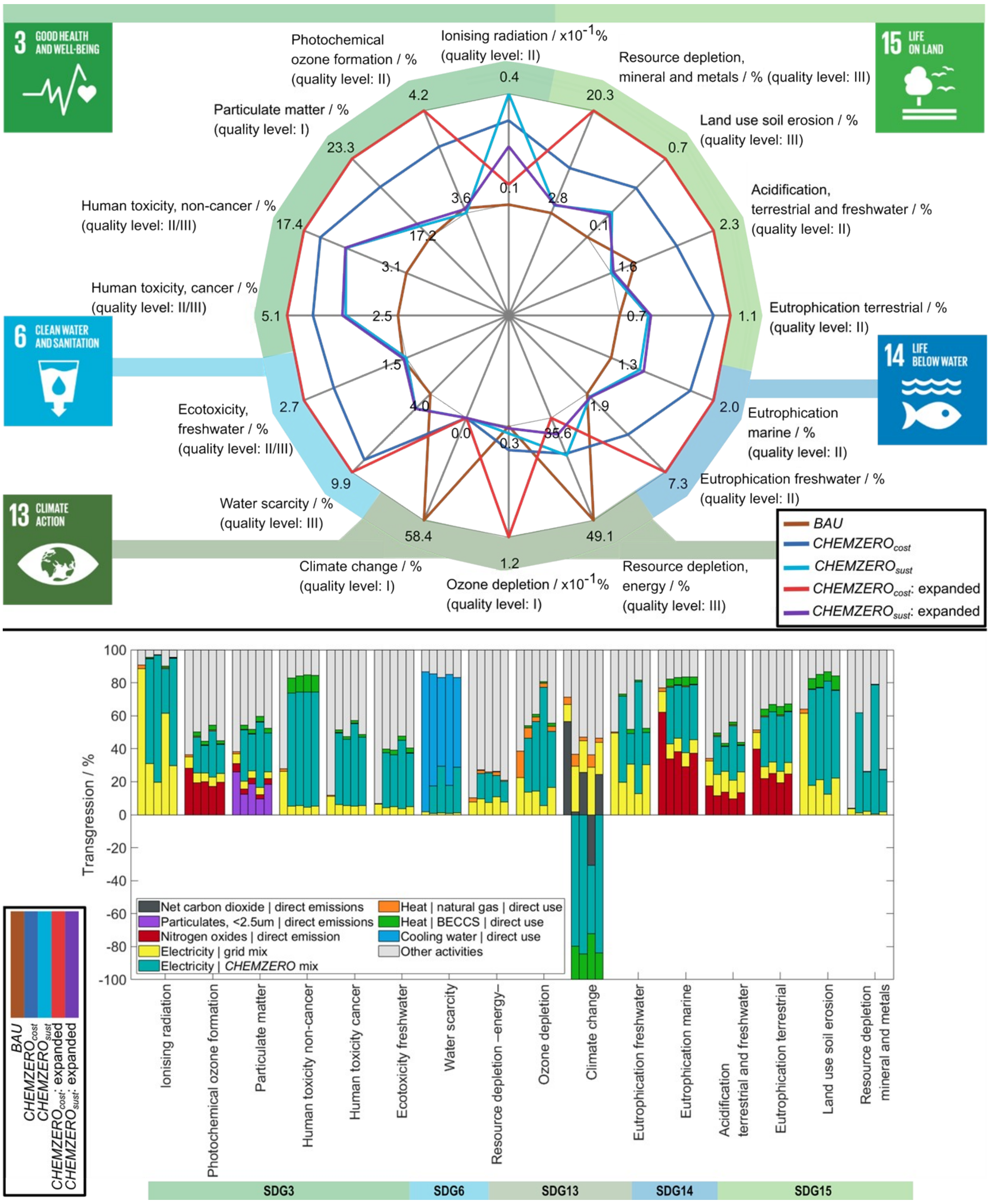

Figure S19. Transgression of the total safe operating space (SOS, i.e., maximum limit defined on the LCA metrics) for the BAU (brown area), $CHEMZERO_{cost}$ (blue area), $CHEMZERO_{sust}$ (cyan area), $CHEMZERO_{cost}^{expanded}$ (red), and $CHEMZERO_{sust}^{expanded}$ (purple) solutions (top), and breakdown of impacts for the latter systems (bottom). Furthermore, the environmental breakdown is presented based on 9 contributors, namely (1) net CO2 emissions, (2) particulate matter (<2.5µm) emissions, (3) nitrogen oxides emissions –where (1), (2), and (3) are emitted directly in the chemical processes–, (4) grid electricity –consumed directly in the conventional chemical processes–, (5) electricity from the CHEMZERO mix, mainly to generate eH_2, (6) heat from natural gas, (7) heat from BECCS –both (6) and (7) are consumed directly in the chemical processes and DAC–, (8) cooling water –consumed directly in the chemical processes–, and (9) other activities, which include inputs of fossil-based resources (or inputs of other nature), and direct emissions to air and water that are not covered in the previously mentioned categories.

Carbon-negative power to produce carbon-neutral chemicals

For the sake of completion, we also provide in **Figure S20** the merit curves for the designs with an expanded portfolio. The expanded portfolio designs lead to almost identical costs relative to their reference counterpart.

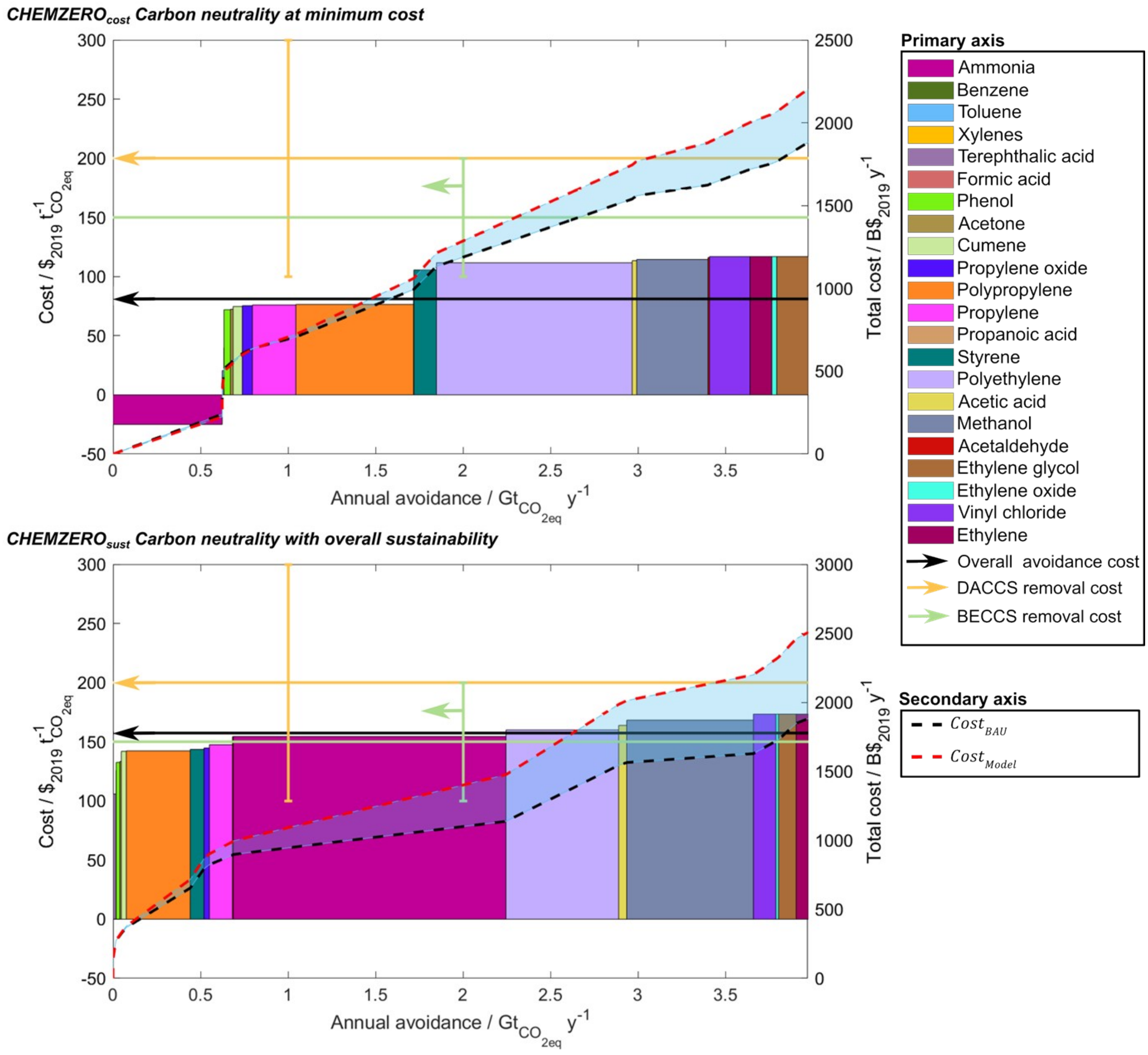

Figure S20. Merit order of chemicals, indicating the CO_2 avoidance cost (primary axis) of each chemical (bars) and the total chemical system (black arrow). In addition, we also demonstrate the forecasted removal cost, rather than the avoidance cost, for DACCS and BECCS (light orange and green arrow, respectively). We further provide the total cost (secondary axis) for the respective solution (red dash line) and the BAU (black dashed line) to meet the 2050's annual avoidance target, equal to 4.0 Gt of CO_{2eq}. The merit order depicts the sequence of the chemical's appeal in the two solutions, replacing the fossil-based pathways entirely, or partially (hybrid), with their renewable alternative to attain a carbon-neutral operation for the chemical system.

3. Model notation

Sets	
i	Product i
j	Technology j
d	Life cycle assessment indicator d
s	Power source s
Variables	
P_i	Purchases of product i
S_i	Sales of product i
TC	Total cost
$TIMP_d$	Total impact on life cycle assessment indicator d
TR_d	Transgression level on life cycle assessment indicator d
TP	Total generated power
W_j	Production rate of technology j
Gen_j^{ST}	Power generated by standard technology j
Gen_j^{BACKUP}	Power generated by backup technology j
CAP_j^{ST}	Capacity of standard power technology j
CAP_j^{BACKUP}	Capacity of standard backup technology j
Parameters	
μ_{ij}	Consumption/production rate of product i with technology j
a_{js}	Indicator of power technology j which generates power with source s
ct_j	Cost of technology j
c_i	Cost of product i
De_i	Demand of product i
PV_j	Production volume of technology j
$IMPT_{jd}$	Impact of technology j in the life cycle assessment indicator d

$IMPP_{id}$	Impact of product i in the life cycle assessment indicator d
SOS_d	Carrying capacity in the life cycle assessment indicator d
CF_j	Capacity factor of power technology j
$CAAGR_s$	Average compound annual growth rate for power technologies using source s
$BACKUP$	Energy system reliability parameter
H	Annual operational hours
$Tech_s^{Pot}$	Global technical potential for generating power with source s
Mix_s^{2021}	Global power generation in 2021 with source s
Mix_s^{2050}	Global power generation in 2050 with source s

4. References

1 J. Kim, S. M. Sen and C. T. Maravelias, *Energy Environ. Sci.*, 2013, **6**, 1093–1104.

2 I. Ioannou, S. C. D'Angelo, A. J. Martín, J. Pérez-Ramírez and G. Guillén-Gosálbez, *ChemSusChem*, 2020, **13**, 6370–6380.

3 A. Galán-Martín, C. Pozo, A. Azapagic, I. E. Grossmann, N. Mac Dowell and G. Guillén-Gosálbez, *Energy Environ. Sci.*, 2018, **11**, 572–581.

4 S. Sala, E. Crenna, M. Secchi and E. Sanyé-Mengual, *J. Environ. Manage.*, 2020, **269**, 110686.

5 S. C. D'Angelo, S. Cobo, V. Tulus, A. Nabera, A. J. Martín, J. Pérez-Ramírez and G. Guillén-Gosálbez, *ACS Sustain. Chem. Eng.*, 2021, **9**, 9740–9749.

6 M. Fasihi, O. Efimova and C. Breyer, *J. Clean. Prod.*, 2019, **224**, 957–980.

7 G. Wernet, C. Bauer, B. Steubing, J. Reinhard, E. Moreno-ruiz and B. Weidema, *Int. J. Life Cycle Assess.*, 2016, **3**, 1218–1230.

8 A. González-Garay, M. S. Frei, A. Al-Qahtani, C. Mondelli, G. Guillén-Gosálbez and J. Pérez-Ramírez, *Energy Environ. Sci.*, 2019, **12**, 3425–3436.

9 Á. Galán-Martín, V. Tulus, I. Díaz, C. Pozo, J. Pérez-Ramírez and G. Guillén-Gosálbez, *One Earth*, 2021, **4**, 565–583.

10 U. S. Energy Information Administration, Levelized Costs of New Generation Resources in the Annual Energy Outlook 2021, https://www.tiny.cc/lixepz, (accessed 5 July 2021).

11 U. S. Energy Information Administration, Levelized Cost and Levelized Avoided Cost of New Generation Resources in the Annual Energy Outlook 2019, https://www.tiny.cc/lixepz, (accessed 5 July 2021).

12 NREL, Electricity annual technology baseline (ATB) data, https://atb.nrel.gov/electricity/2021/data.

13 F. Petrakopoulou, D. Iribarren and J. Dufour, *Greenh. Gases Sci. Technol.*, 2015, **5**, 268–276.

14 C. Wildbolz, Swiss Federal Institute of Technology Zurich, 2007.

15 D. Iribarren, F. Petrakopoulou and J. Dufour, *Energy*, 2013, **50**, 477–485.

16 J. Koornneef, T. van Keulen, A. Faaij and W. Turkenburg, *Int. J. Greenh. gas Control*, 2008, **2**, 448–467.

17 G. D. Oreggioni, B. Singh, F. Cherubini, G. Guest, C. Lausselet, M. Luberti, H. Ahn and A. H. Strømman, *Int. J. Greenh. Gas Control*, 2017, **57**, 162.

18 L. A.-W. Ellingsen, G. Majeau-Bettez, B. Singh, A. K. Srivastava, L. O. Valøen and A. H. Strømman, *J. Ind. Ecol.*, 2014, **18**, 113–124.

19 O. Edenhofer, R. Pichs-Madruga, Y. Sokona, K. Seyboth, S. Kadner, T. Zwickel, P. Eickemeier, G. Hansen, S. Schlömer and C. von Stechow, *Renewable energy sources*

and climate change mitigation: Special report of the intergovernmental panel on climate change, Cambridge University Press, 2011.

20 IEA, World Energy Outlook 2019, IEA, Paris, https://www.iea.org/reports/world-energy-outlook-2019.

21 G. Iyer, N. Hultman, J. Eom, H. McJeon, P. Patel and L. Clarke, *Technol. Forecast. Soc. Change*, 2015, **90**, 103–118.

22 IEA, *Technology Roadmap - Energy and GHG Reductions in the Chemical Industry via Catalytic Processes*, Paris, 2013, vol. 56.

23 Global propionic acid market, https://www.grandviewresearch.com/press-release/global-propionic-acid-market, (accessed 5 July 2021).

24 M. Aresta, A. Dibenedetto and E. Quaranta, *J. Catal.*, 2016, **343**, 2–45.

25 Global Acetaldehyde Market, https://www.prweb.com/releases/acetaldehyde/acetic_ether_pyridine/prweb8070299.htm, (accessed 5 July 2021).

26 Global acetic acid market, https://www.credenceresearch.com/press/global-acetic-acid-market, (accessed 5 July 2021).

27 S. Searle and C. Malins, *GCB Bioenergy*, 2015, **7**, 328–336.

28 G. Doka, *Combining life cycle inventory results with planetary boundaries: the planetary boundary allowance impact assessment method PBA'05*, Zurich, Switzerland, 2015.

29 *European Commission - Joint Research Centre - Institute of Environment and Sustainability. Supporting information to the characterisation factors of recommended EF Life Cycle Impact Assessment methods: New methods and differences with ILCD*, Publications Office of the European Union, Luxembourg, 2018.

30 *European Commission-Joint Research Centre - Institute for Environment and Sustainability: International Reference Life Cycle Data System (ILCD) Handbook-Recommendations for Life Cycle Impact Assessment in the European context*, Publications Office of the European Union, Luxemburg, 2011.

31 IEA (2020), Projected Costs of Generating Electricity 2020, IEA, Paris, https://www.iea.org/reports/projected-costs-of-generating-electricity-2020.

32 P. L. Lucas, H. C. Wilting, A. F. Hof and D. P. van Vuuren, *Glob. Environ. Chang.*, 2020, **60**, 102017.

33 A. Bjorn, C. Chandrakumar, M. B. Anne, G. Doka, K. Fang, N. Gondran, Z. H. Michael, A. Kerkhof, H. King, M. Margni, S. McLaren, C. Mueller, M. Owsianiak, G. Peters, S. Roos, S. Sala, G. Sandin, S. Sim, M. Vargas-Gonzalez and R. Morten, *Environ. Res. Lett.*, 2020, **15**, 83001.

34 A. Bjørn and M. Z. Hauschild, *Int. J. Life Cycle Assess.*, 2015, **20**, 1005–1018.

35 P. Breeze, ed. P. B. T.-C. H. and P. Breeze, Academic Press, 2018, pp. 41–49.

36 P. J. First, *Sustain. Dev. Efforts to Eradicate Poverty. https//www. ipcc. ch/sr15/. Accessed*.

37 K. Anderson and G. Peters, *Science*, 2016, **354**, 182–183.

38 D. Lenzi, *Glob. Sustain.*, 2018, **1**, E7.

39 Data from United Nations (UN) Commodity Trade Statistics Database, https://data.un.org/, (accessed 5 July 2021).

40 A. Mehmeti, A. Angelis-Dimakis, G. Arampatzis, S. J. McPhail and S. Ulgiati, *Environments*, 2018, **5**, 24.

41 R. Turton, R. C. Bailie, W. B. Whiting and J. A. Shaeiwitz, *Analysis, synthesis and design of chemical processes*, Pearson Education, 2008.

42 Y. Mujaj, Association for Computing Machinery, New York, 2008.

43 R. Li, C.-K. Woo, A. Tishler and J. Zarnikau, *Util. Policy*, 2022, **74**, 101318.

44 J. C. B. Cooper, *OPEC Rev.*, 2003, **27**, 1–8.

45 M. Jussila, S. Tamminen and J. Kinnunen, *The estimation of LES demand elasticities for CGE models*, Valtion taloudellinen tutkimuskeskus VATT, Helsinki, 2012.

46 M. A. QUDDUS, M. W. SIDDIQI and M. M. RIAZ, *Pak. Econ. Soc. Rev.*, 2008, **46**, 101–116.

47 C. Chatterton, Methanol as an alternative fuel, https://www.methanol.org/wp-content/uploads/2018/10/MI-Asian-Sulphur-Cap-2020-Final.pdf, (accessed 1 June 2022).

48 M. Corporation, Methanol prices for North America, Europe and Asia, https://www.methanex.com/our-business/pricing, (accessed 1 June 2022).

49 World GDP Per Capita 1960-2022, https://www.macrotrends.net/countries/WLD/world/gdp-per-capita, (accessed 1 June 2022).

50 World inflation rate, https://www.macrotrends.net/countries/WLD/world/inflation-rate-cpi, (accessed 1 June 2022).
